# Supplementary material for: T-Cell-Specific Deletion of Map3k1 Reveals the Critical Role for Mekk1 and Jnks in Cdkn1b-Dependent Proliferative Expansion
Source: Cell Rep. 2016 Jan 7;14(3):449–57. doi: 10.1016/j.celrep.2015.12.047 (PMC4733086; doi:10.1016/j.celrep.2015.12.047)
Supplement: Document S1. Supplemental Experimental Procedures, Figures S1–S5, and Tables S1–S3 [file mmc1.pdf]

Cell Reports
Supplemental Information

T-Cell-Specific Deletion of *Map3k1* Reveals the Critical Role for Mekk1 and Jnks in *Cdkn1b*-Dependent Proliferative Expansion

Tesha Suddason, Saba Anwar, Nikolaos Charlaftis, and Ewen Gallagher

Supplementary information

Supplementary materials

Mice

Map3k1$^{\Delta KD}$ mice were generated as previously described (Gao et al., 2004), and *Cdkn1b*$^{-/-}$ mice (The Jackson Laboratory) were maintained in accordance with the vendor's protocols. To produce mice with a T cell-specific *Map3k1* deletion, *Map3k1*$^{f/f}$ homozygote mice were crossed with *Lck*^{Cre} mice (Jackson Laboratory) (Hennet et al., 1995). All strains of mice were present on the C57BL/6 background, and bred and maintained under pathogen free conditions in accordance with the guidelines of Imperial College London and the Home Office (London, UK).

Reagents and antibodies

PBS-57-loaded CD1d tetramers were provided by the National Institutes of Health (NIH) Tetramer Facility (Atlanta, USA). KRN7000 (Enzo Life Sciences), an α-GalCer analogue, was used to stimulate iNKT cells. Anti-APC TCRβ (H57-597) (eBioscience), anti-FITC CD3ε (145-2C11) (eBioscience), anti-FITC NK1.1 (PK136) (eBioscience), anti-APC CD44 (IM7) (eBioscience), anti-FITC CD8 (53-6.7) (eBioscience), anti-PE Cy7-CD4 (GK1.5) (eBioscience), anti-FITC γδ TCR (eBioscience) anti-PE Cy7-IFN-γ (BVD6-24G2) (Cell Signaling), anti-phospho p38 (15) (Cell Signaling), anti-phospho JNK (26) (Cell Signaling), anti-p27^{Kip1} antibody (JZA0211031) (R&D Systems), anti-HA (32-6700) (Invitrogen), anti-Myc (R950-25) (Invitrogen), anti-p38 (9212) (Cell Signaling), anti-phospho p38 (9211) (Cell Signaling), anti-JNK (9252) (Cell Signaling), anti-c-Jun (60A8) (Cell Signaling), anti-phospho c-Jun (Ser73) (D47G9) (Cell Signaling), anti-phospho c-Jun (Ser73) (D47G9) XP® Rabbit mAb (Alexa Fluor® 488 Conjugate) (Cell Signaling), anti-

CARD11 (sc-20458) (Cell Signaling), anti-mouse Ub (13-1600) (Invitrogen), anti-Lys63-linked Ub (5621) (Cell Signaling), anti-phospho ERK (M8159) (Sigma), anti-ERK (M5670) (Sigma) and anti-Tubulin (T5168) (Sigma) antibodies were used according to the manufacturers protocols. SP600125 (IC_{50} 40 nM JNK inhibitor, Sigma), PD98059 (IC_{50} 2 μM MEK inhibitor, Sigma), SB203580 (IC_{50} 0.5 μM p38 inhibitor, Sigma), SU9516 (IC_{50} 200 nM CDK inhibitor, Santa Cruz) and NSC697923 (IC_{50} 745 nM Ube2N inhibitor, Millipore) were used according to the manufacturers protocols. Mekk1 constructs were generated and utilized as previously described (Charlaftis et al., 2014).

Supplementary figure and table legends

Figure S1. T cell-specific deletion of *Map3k1*, related to figure 1. (A) Schematic illustrating the genomic PCR genotyping strategy for the floxed *Map3k1* allele. (B) Genomic PCR of the floxed *Map3k1* allele (WT band of 266 bp, mutant band of 113 bp and recombinant band of 408 bp) using DNA prepared from WT, *Map3k1*$^{f/+}$ and *Map3k1*$^{f/f}$ mice. (C) $CD4^+$ T cells were isolated from WT, *Map3k1*$^{\Delta KD}$ and *Lck*$^{Cre/+}$ *Map3k1*$^{f/f}$ mice and incubated with anti-CD3 and anti-CD28 antibodies. Cytokine mRNA level was quantitated after 24 hrs by real-time PCR and the relative amount of *Il4* mRNA to WT cells was given an arbitrary value of 1. The average relative amount (± SEM) from 3 independent experiments was statistically analyzed by two-tailed Student's t test (*, $p \leq 0.05$; **, $p \leq 0.01$; ***, $p \leq 0.001$). (D) Intraepithelial lymphocytes were isolated from the small intestine of WT and *Lck*$^{Cre/+}$ *Map3k1*$^{f/f}$ mice, stained with anti-γδ TCR and anti-CD3 antibodies and analyzed by flow cytometry as indicated. Data is representative of 3 independent experiments. Numbers in the profiles indicate the percentages of the gated populations. (E) Thymocytes,

splenocytes and liver cells from WT and *Lck*$^{Cre/+}$ mice were isolated, stained with anti-CD4 and anti-CD8 antibodies and analyzed by flow cytometry as indicated. Data is representative of 3 independent experiments. Numbers in the profiles indicate the percentages of the gated populations.

Figure S2. *Map3k1* regulates iNKT cells, related to figure 2. (A) Cell suspensions were isolated from the spleen, thymus, bone marrow and liver from WT and *Lck*$^{Cre/+}$ mice, stained with CD1d tetramer and anti-CD3 antibodies and analyzed by flow cytometry as indicated. Data is representative of 3 independent experiments. Numbers in the profiles indicate the percentages of the gated populations. (B) Splenocyte, thymocyte, bone marrow and liver cells from WT, *Map3k1*$^{\Delta KD}$ and *Lck*$^{Cre/+}$*Map3k1*$^{f/f}$ mice were isolated, stained with PBS-57-loaded CD1d tetramer and anti-NK1.1 antibody and analyzed by flow cytometry as indicated. Data is representative of 3 independent experiments. Numbers in the profiles indicate the percentages of the gated populations. (C) Statistical analysis of the iNKT population (CD1d tetramer^{+}TCRβ^{+}) within the thymus from WT, *Map3k1*$^{\Delta KD}$ and *Lck*$^{Cre/+}$ *Map3k1*$^{f/f}$ mice. The average percentage (± SEM) of CD1d-tetramer and TCRβ positive cells from 5 independent experiments is shown (• WT, ■ *Map3k1*$^{\Delta KD}$ and ▲ *Lck*$^{Cre/+}$ *Map3k1*$^{f/f}$ mice). Statistical differences were analyzed by two-tailed Student's t test (*, $p \leq 0.05$; **, $p \leq 0.01$; ***, $p \leq 0.001$).

Figure S3. Analysis of Mekk1 signaling in iNKT cells, related to figure 3. (A) Quantitation of phospho-Jnk activation in Fig. 3A. Mean intensity for each experimental point was measured by densitometry. (B) Quantitation of phospho-p38 activation in Fig. 3A. Mean intensity for each experimental point was measured by

densitometry. (C) iNKT cells were isolated (4 mice per experiment) and stimulated by TCR crosslinking with antibodies over a 60 mins timecourse. Lysates were made under non-denaturing conditions and IB and IP performed as indicated. (D) A model illustrating Mekk1-dependent TCR signaling in iNKT cells.

Figure S4. Role of *Map3k1* in the iNKT cell response to glycolipid antigen, related to figure 4. (A) Short-term iNKT cell expansion in WT and $Lck^{Cre/+}$ $Map3k1^{f/f}$ mice. WT (-) and $Lck^{Cre/+}$ $Map3k1^{f/f}$ (-) mice were i.p. immunized with α-GalCer for 2 hrs, splenocytes isolated and stained with CD1d tetramer, anti-IFN-γ (top panel), anti-IL-4 (middle panel) and anti-CD69 (bottom panel) antibodies and analyzed by flow cytometry as indicated. Data is representative of three independent experiments (2 mice per experiment). Histograms show the IFN-γ (top panel), IL-4 (middle panel) and CD69 (bottom panel) present in the gated iNKT cell population. (B) WT, $Map3k1^{\Delta KD}$ and $Lck^{Cre/+}$ $Map3k1^{f/f}$ mice were treated with water containing BrdU and i.p. immunized with α-GalCer for 3 days or left unimmunized. iNKT cells were isolated as above (■ spleen and ■ liver) and analyzed by flow cytometry for BrdU incorporation. Representative results from 3 quantitated iNKT proliferation experiments were statistically analyzed, where appropriate, by two-tailed Student's t test (*, $p \leq 0.05$; **, $p \leq 0.01$; ***, $p \leq 0.001$). (C) Statistical analysis of α-GalCer-dependent liver iNKT expansion at days 0 and 3 in *Map3k1*-deficient mice. The average percentage (± SEM) of PBS-57-loaded CD1d tetramer$^+$ TCRβ$^+$ cells from 5 independent experiments is shown (■ WT, ■ $Map3k1^{\Delta KD}$ and ■ $Lck^{Cre/+}$ $Map31^{f/f}$ mice). Differences were analyzed by two-tailed Student's t test (*, $p \leq 0.05$; **, $p \leq 0.01$; ***, $p \leq 0.001$). (D) Representative H&E stained liver sections were prepared from unstimulated (upper panels) and 3-day α-GalCer stimulated (lower panels) WT

and $Lck^{Cre/+}$ mice (original magnification x40, scale bar = 10 μM). Data is representative of 3 independent experiments (2 mice per experiment).

Figure S5. Mekk1 signaling regulates *Cdkn1b* expression and iNKT cell proliferative expansion, related to figure 5. (A) The bioinformatics gene wheel analyzes global gene expression hits from $Map3k1^{\Delta KD}$ iNKT cells and indicates their cellular and molecular function. Color intensity indicates the range of expression from an upregulation of 3.347 to a downregulation of -5.847. (B) WT and $Lck^{Cre/+}$ $Map3k1^{f/f}$ mice were 3-day immunized with α-GalCer (■ WT and ■ $Lck^{Cre/+}$ $Map3k1^{f/f}$), RNA was extracted from their splenic iNKT cells, reverse transcribed and real-time PCR performed. The average relative expression (± SEM) of genes from 3 independent experiments was statistically analyzed, where appropriate, by two-tailed Student's t test (*, $p \le 0.05$; **, $p \le 0.01$; ***, $p \le 0.001$). (C) WT and $Lck^{Cre/+}$ $Map3k1^{f/f}$ mice were 3-day immunized with α-GalCer, RNA was extracted from liver iNKT cells, RNA was reverse transcribed and real-time PCR performed (■ WT and ■ $Lck^{Cre/+}$ $Map3k1^{f/f}$). The average relative expression (± SEM) of genes from 3 independent experiments was statistically analyzed, where appropriate, by two-tailed Student's t test (*, $p \le 0.05$; **, $p \le 0.01$; ***, $p \le 0.001$). (D). RNA was isolated from WT and $Map3k1^{\Delta KD}$ iNKT cells, processed and hybridized onto Affymetrix arrays. Bioinformatics analysis was performed on integrin gene expression as indicated and a heat map comparing gene hits between WT and $Map3k1^{\Delta KD}$ iNKT cell microarray screens was constructed. The data is from 3 independent experiments (4 mice per experiment).

Table S1. Analysis of thymocytes in *Map3k1* mutant mice, related to figure 1. Total numbers of thymocytes from WT and *Lck*$^{Cre/+}$ *Map3k1*$^{f/f}$ mice were counted and percentages of subpopulations were quantitated by FACS analysis (Gao et al., 2004).

	**WT**	***Lck*$^{Cre/+}$ *Map3k1*$^{f/f}$ mice**
**Total cell number (x10^6)**	172 ±49	175 ±51
**$CD4^+CD8^+$(%)**	91.65±0.30	86.1±0.808
**$CD4^-CD8^+$(%)**	6.28±0.204	10.56±1.073
**$CD4^+CD8^-$(%)**	10.93±0.052	14.4±0.221

Table S2. iNKT cell affymetrix microarray hits from *Map3k1*-deficient versus control mice undergoing a clonal burst (2-fold or more changes are listed), related to figure 5.

ID	Fold Change	Entrez Gene Name	Location	Type(s)
Klrc3	2.799	killer cell lectin-like receptor subfamily C, member 2	Plasma Membrane	other
Ar	2.397	androgen receptor	Nucleus	ligand-dependent nuclear receptor
Klrk1	2.266	killer cell lectin-like receptor subfamily K, member 1	Plasma Membrane	transmembrane receptor
Klk1b11	2.186	kallikrein-related peptidase 3	Extracellular Space	peptidase
Cd101	-2.008	CD101 molecule	Plasma Membrane	other
Cd177	-2.039	CD177 molecule	Cytoplasm	other
Clec4d	-2.042	C-type lectin domain family 4, member D	Plasma Membrane	other
Il1r2	-2.054	interleukin 1 receptor, type II	Plasma Membrane	transmembrane receptor
Rorc	-2.066	RAR-related orphan receptor C	Nucleus	ligand-dependent nuclear receptor
Cd33	-2.078	CD33 antigen	Plasma Membrane	other
Tdgf1	-2.110	teratocarcinoma-derived growth factor 1	Extracellular Space	growth factor
Il1b	-2.152	interleukin 1, beta	Extracellular Space	cytokine
Camp	-2.230	cathelicidin antimicrobial peptide	Cytoplasm	other
Csf3r	-2.246	colony stimulating factor 3 receptor (granulocyte)	Plasma Membrane	transmembrane receptor
Cxcr2	-2.257	chemokine (C-X-C motif) receptor 2	Plasma Membrane	G-protein coupled receptor
Il17rb	-2.345	interleukin 17 receptor B	Plasma Membrane	transmembrane receptor
Mmp9	-2.345	matrix metallopeptidase 9 (gelatinase B, 92kDa gelatinase, 92kDa type IV collagenase)	Extracellular Space	peptidase
Cdkn1b	-2.386	cyclin-dependent kinase inhibitor 1B (p27, Kip1)	Nucleus	kinase
Retnla	-2.628	resistin like alpha	Extracellular Space	other
Rbm39	-2.672	RNA binding motif protein 39	Nucleus	transcription regulator
Il1f9	-2.699	interleukin 36, gamma	Extracellular Space	cytokine
Rapgef1	-2.914	Rap guanine nucleotide exchange factor (GEF) 1	Cytoplasm	other
Ptger1	-3.025	prostaglandin E receptor 1 (subtype EP1), 42kDa	Plasma Membrane	G-protein coupled receptor

Table S3. Listing of the primers used for real-time PCR, related to experimental procedures.

	5' to 3'	
	Forward Primer	Reverse Primer
β-actin	GTCGACAACGGCTCCGGCATGT	TCCCACCATCACACCCTGGTGCCTA
Ar	TGAGCCAGGAGTGGTGTGTGC	AAGTTGCGGAAGCCAGGCAAGG
Camp	AGGAACAGGGGGTGGTGAAGCA	AGAAGTCCAGCCAGCCGGGAA
Cdkn1b	GCCAGACGTAAACAGCTCCGAATTA	AGAGGCAGATGGTTTAAGAGTGCCT
CD101	AGTTTCTGCTAAGTTCAGCATCGGC	TTCCTTGGTCGGGGCGCTTG
CD177	CCTTGCTACCCTGTGTCCCAGC	GGCAACCCTCGCTAACCTCGC
CD33	GGGGAGGCAACGGTCAAGCTC	CCTGCTGATGAGCCTGTGTATGGAA
Clec4d	ACCCGACATCCCCAACTGATCCC	GCAGGTCCAAGTACCTCCTGTAGC
Csf3r	ACCCCATGGATGTTGCCCCC	CTTCCTGCAGGGGCGTTGGC
Cxcr2	ATCTTCGCTGTCGTCCTTGT	AGCCAAGAATCTCCGTAGCA
Il17rb	GGAGGCAAGGAAGGAGCACGA	CGGCCCCATCTCGGCGATTT
Il1b	CCCTGCAGCTGGAGAGTGTGGA	TGTGCTCTGCTTGTGAGGTGCTG
Il1f9	CCACAGAGTAACCCCAGTCAGCG	TTCCACCTGTCCGGGTGTGGT
Il1r2	CGGGTCAAAGGAACAACCACGGA	CGGTCACACGGCCTCTTGGG
klk1b11	CACCCACGAAATTCCAAACCCCAG	CCGGCGTATTGGGTTTGCCACA
klrc3	AGACAGTGAAGAGGAGCAGGACTTT	TCTGCTGTGAGACCAGAAGCTGAC
klrk1	TCTGCTCAGAGATGAGCAAATGCC	GCCAAGGCTATAGCAAGGACTCGAA
Mmp9	TGCCCTACCCGAGTGGACGC	AGCCCAGTGCATGGCCGAAC
Ppbp	TCCTTGTTGCGCTGGCTCCC	GTGTGGCTATCACTTCCACATCAGC
Ptger1	ACATGCATGGGGTGGAGCAGC	TATCAGTGGCCAAGAGGGCCAG
Retnla	CCTGCCCTGCTGGGATGACT	GGGCAGTGGTCCAGTCAACGA
Rorc	TTCCCACTTCCTCAGCGCCC	TGGGTGGCAGCTTGGCTAGGA
Tdgf1	TCGCAAAGAGCACTGTGGGTC	AGTGGTCGTCACAGACGGCG

Supplementary references

CHARLAFTIS, N., SUDDASON, T., WU, X., ANWAR, S., KARIN, M. & GALLAGHER, E. 2014. The MEKK1 PHD ubiquitinates TAB1 to activate MAPKs in response to cytokines. *EMBO J,* 33**,** 2581-96.

GAO, M., LABUDA, T., XIA, Y., GALLAGHER, E., FANG, D., LIU, Y. C. & KARIN, M. 2004. Jun turnover is controlled through JNK-dependent phosphorylation of the E3 ligase Itch. *Science,* 306**,** 271-5.

HENNET, T., HAGEN, F. K., TABAK, L. A. & MARTH, J. D. 1995. T-cell-specific deletion of a polypeptide N-acetylgalactosaminyl-transferase gene by site-directed recombination. *Proc Natl Acad Sci U S A,* 92**,** 12070-4.

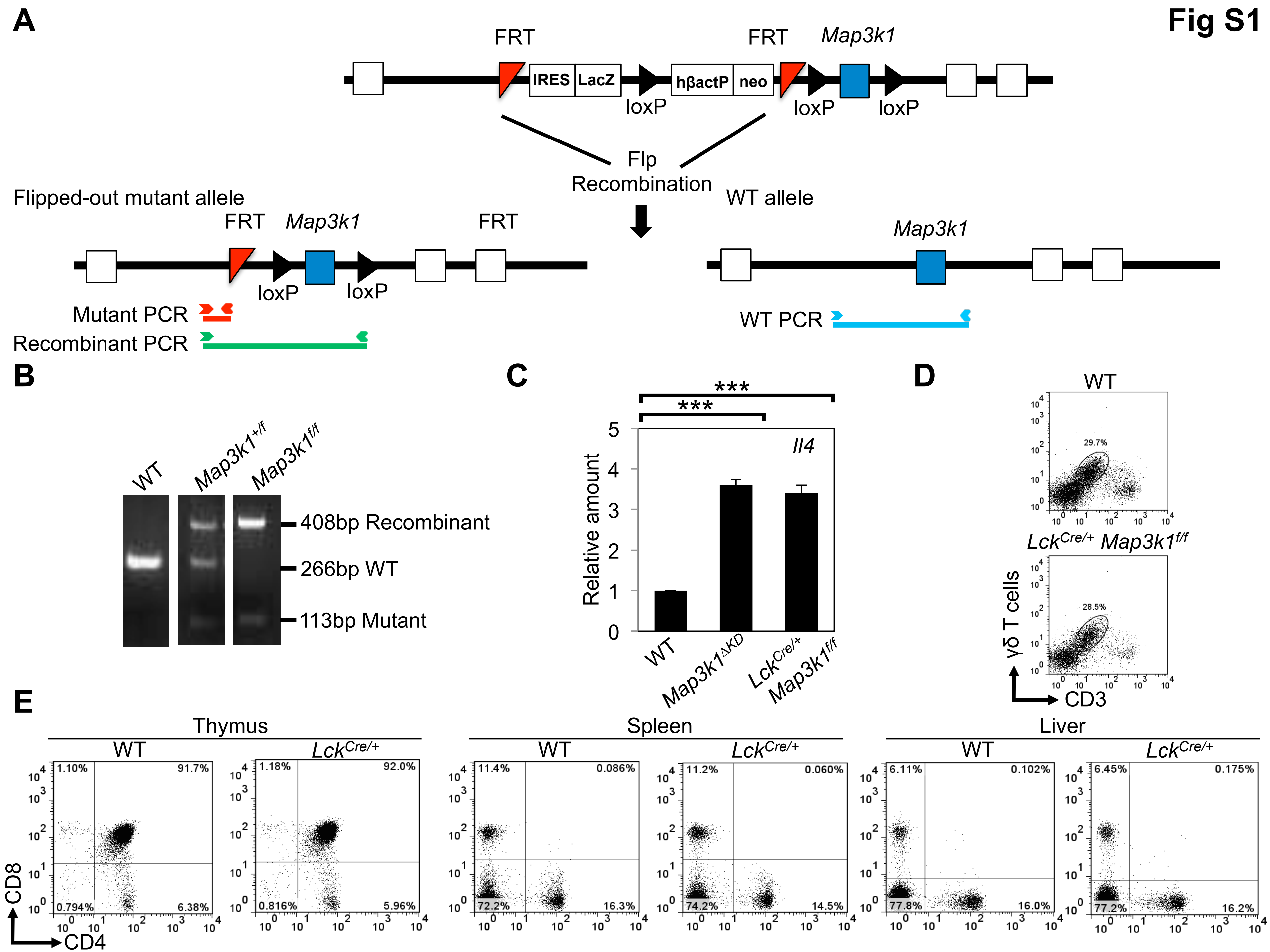
Fig S1
A
FRT
IRES
LacZ
loxP
hβactP
neo
FRT
loxP
Map3k1
loxP
Flp
Recombination
Flipped-out mutant allele
FRT
Map3k1
FRT
loxP
loxP
Mutant PCR
Recombinant PCR
WT allele
Map3k1
WT PCR
B
WT
Map3k1+/f
Map3k1f/f
408bp Recombinant
266bp WT
113bp Mutant
C
***
***
Il4
Relative amount
WT
Map3k1ΔKD
LckCre/+ Map3k1f/f
D
WT
29.7%
LckCre/+ Map3k1f/f
28.5%
γδ T cells
CD3
E
Thymus
Spleen
Liver
WT
LckCre/+
1.10%
91.7%
0.794%
6.38%
1.18%
92.0%
0.816%
5.96%
11.4%
0.086%
72.2%
16.3%
11.2%
0.060%
74.2%
14.5%
6.11%
0.102%
77.8%
16.0%
6.45%
0.175%
77.2%
16.2%
CD8
CD4

Fig S2

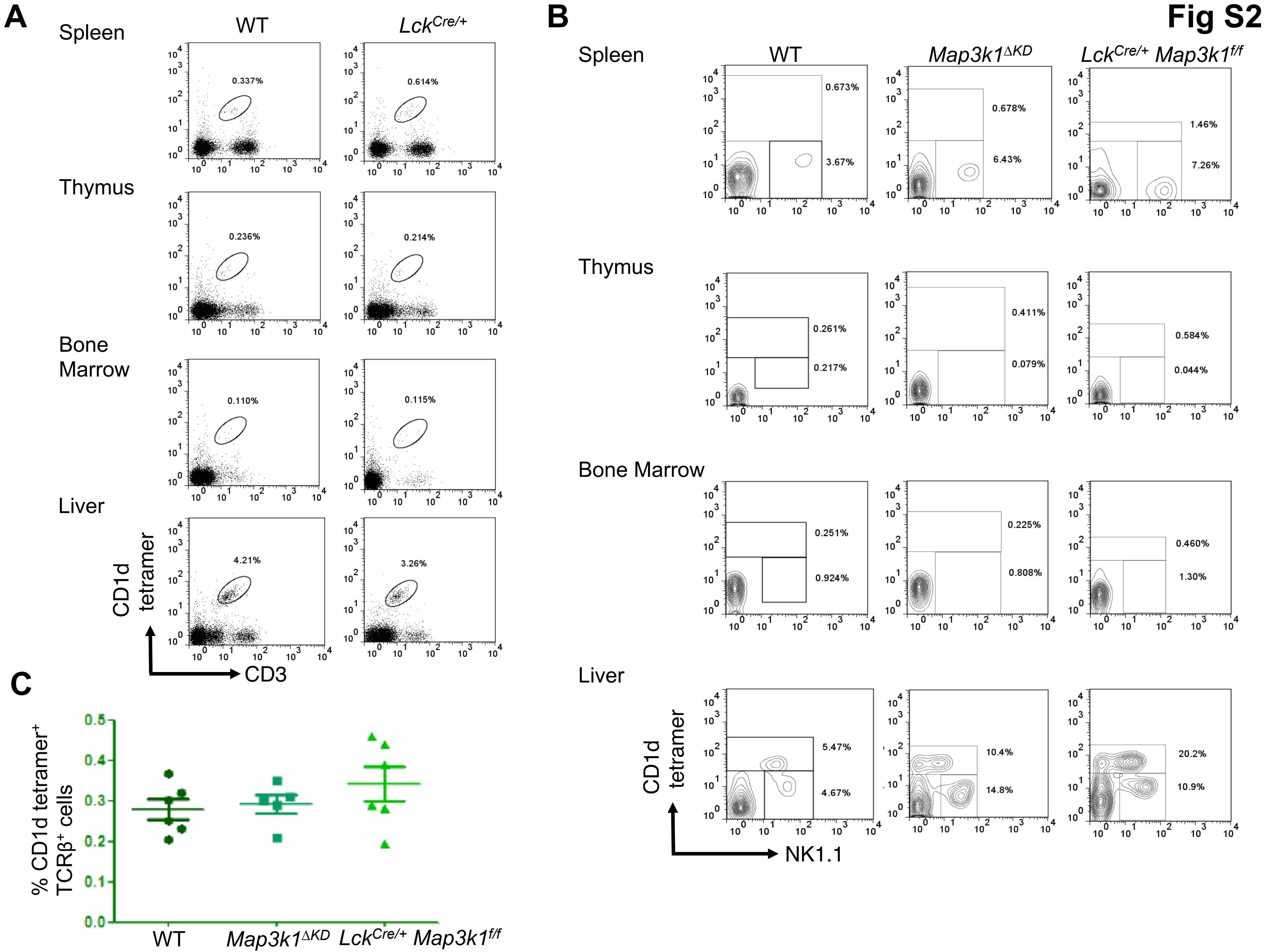
A
Spleen
Thymus
Bone Marrow
Liver
WT
LckCre/+
0.337%
0.614%
0.236%
0.214%
0.110%
0.115%
4.21%
3.26%
CD1d tetramer
CD3
B
Spleen
Thymus
Bone Marrow
Liver
WT
Map3k1ΔKD
LckCre/+ Map3k1f/f
0.673%
3.67%
0.678%
6.43%
1.46%
7.26%
0.261%
0.217%
0.411%
0.079%
0.584%
0.044%
0.251%
0.924%
0.225%
0.808%
0.460%
1.30%
5.47%
4.67%
10.4%
14.8%
20.2%
10.9%
CD1d tetramer
NK1.1
C
% CD1d tetramer+ TCRβ+ cells
0.0
0.1
0.2
0.3
0.4
0.5
WT
Map3k1ΔKD
LckCre/+ Map3k1f/f

Fig S3

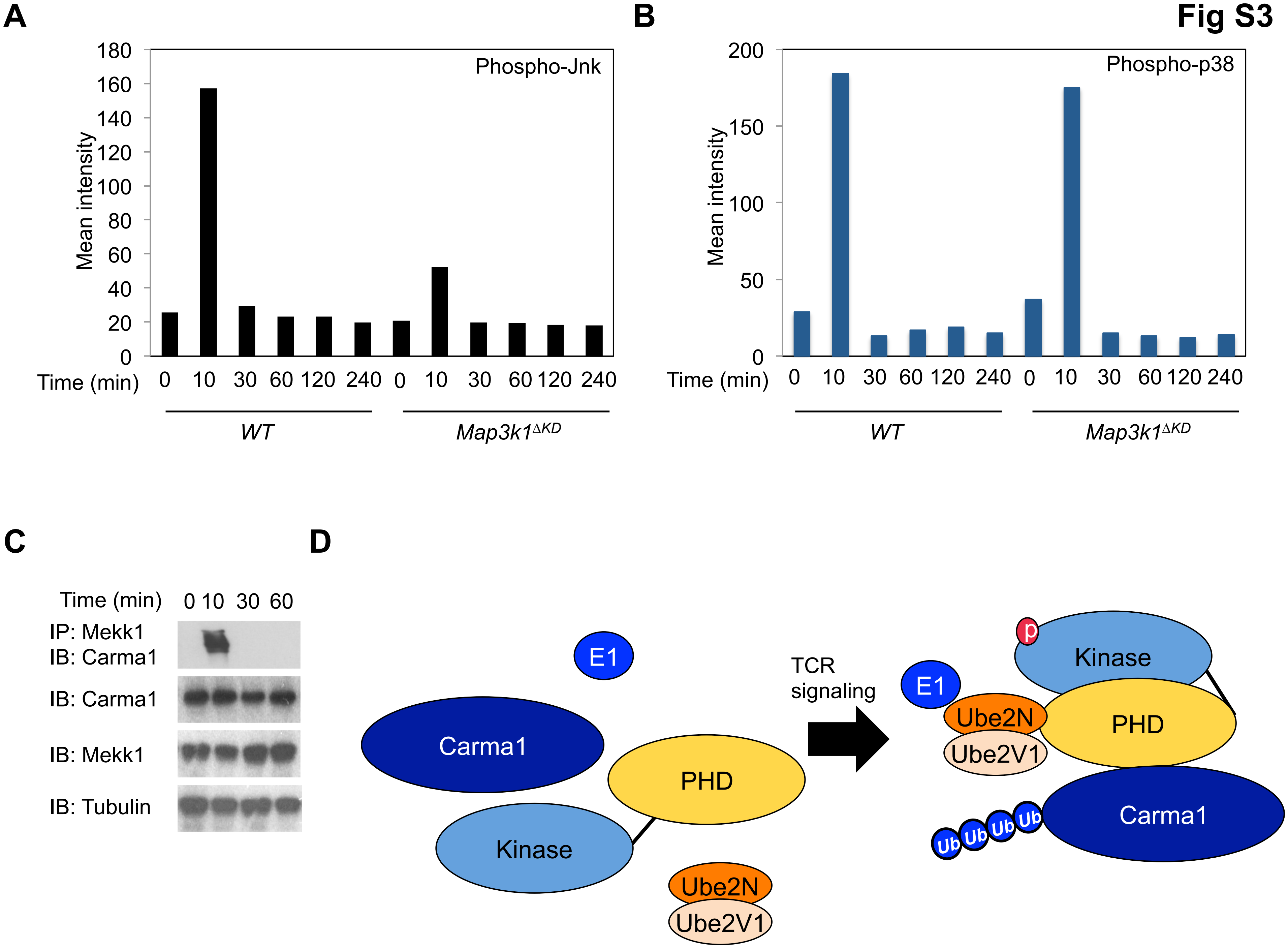

A
Phospho-Jnk
Mean intensity
180
160
140
120
100
80
60
40
20
0
Time (min)
0 10 30 60 120 240 0 10 30 60 120 240
WT
Map3k1ΔKD
B
Phospho-p38
Mean intensity
200
150
100
50
0
Time (min)
0 10 30 60 120 240 0 10 30 60 120 240
WT
Map3k1ΔKD
C
Time (min)
0 10 30 60
IP: Mekk1
IB: Carma1
IB: Carma1
IB: Mekk1
IB: Tubulin
D
E1
Carma1
PHD
Kinase
Ube2N
Ube2V1
TCR
signaling
p
Kinase
E1
Ube2N
PHD
Ube2V1
Carma1
Ub Ub Ub Ub

Fig S4

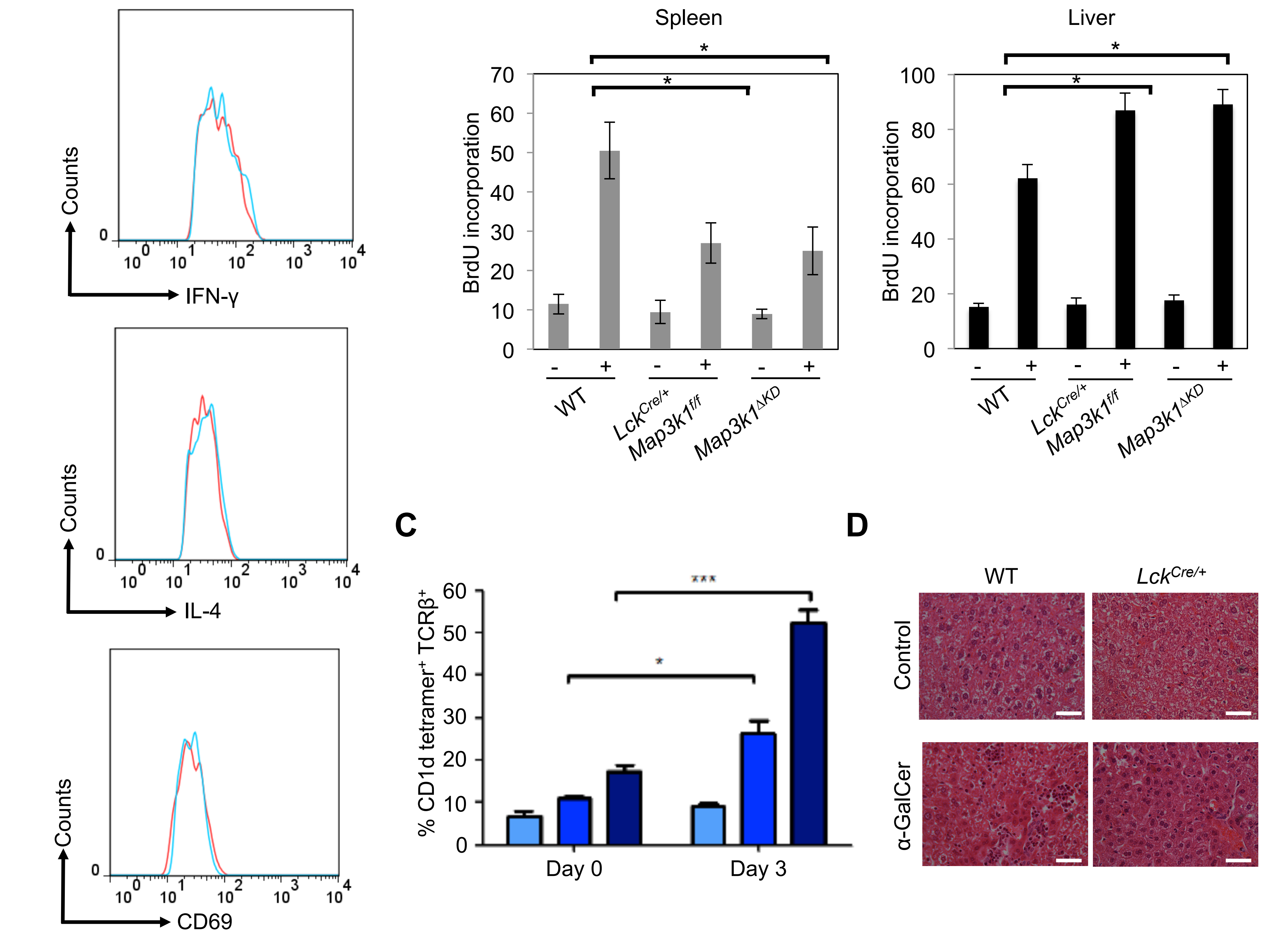
A
Counts
IFN-γ
IL-4
CD69
B
Spleen
Liver
BrdU incorporation
*
WT
LckCre/+ Map3k1f/f
Map3k1ΔKD
C
% CD1d tetramer+ TCRβ+
***
*
Day 0
Day 3
D
WT
LckCre/+
Control
α-GalCer

Fig S5

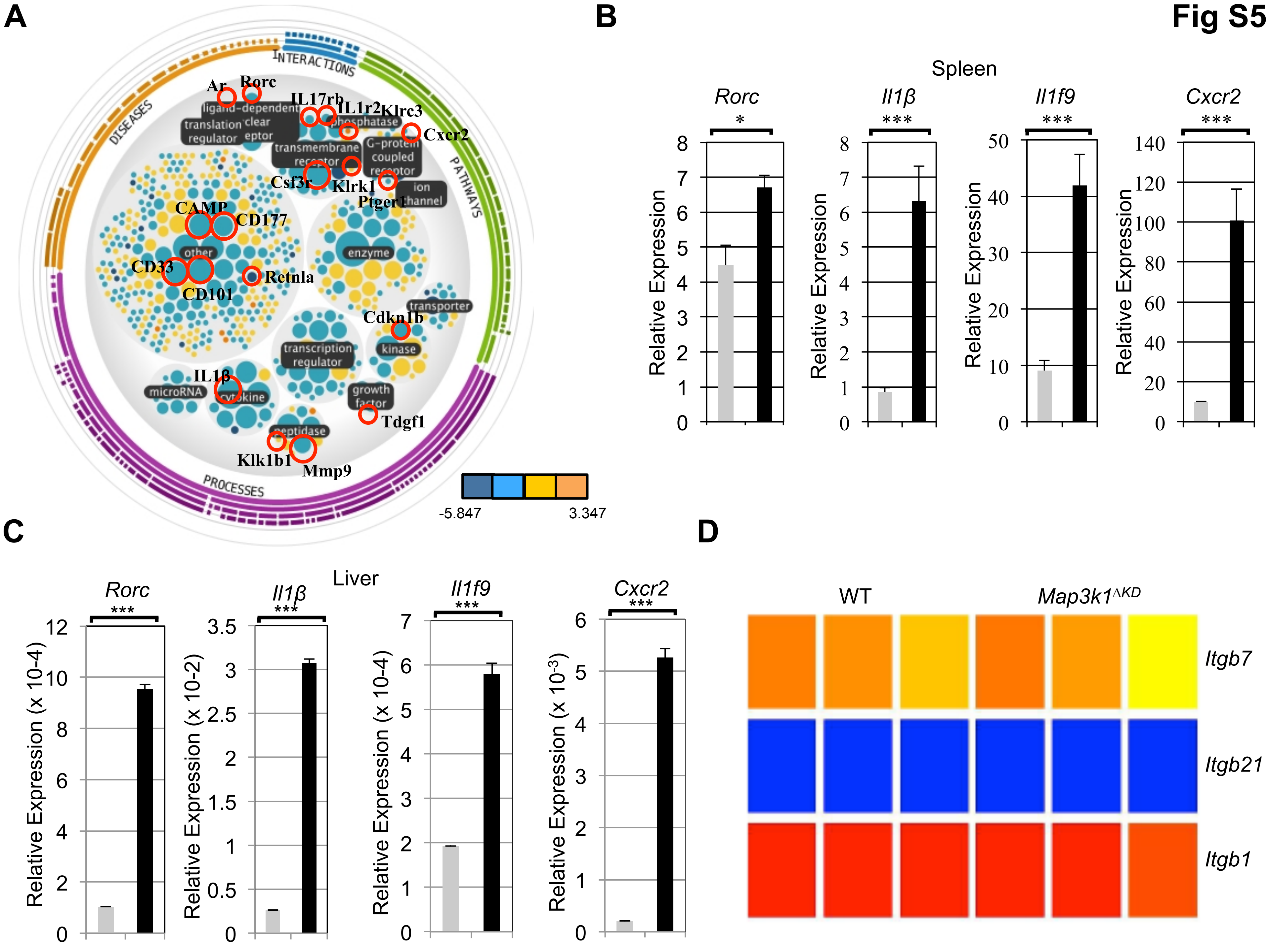
A
INTERACTIONS
DISEASES
PATHWAYS
PROCESSES
Ar
Rorc
IL17rb
IL1r2
Klrc3
Cxcr2
Csf3r
Klrk1
Ptger1
CAMP
CD177
CD33
CD101
Retnla
Cdkn1b
IL1β
Tdgf1
Klk1b1
Mmp9
ligand-dependent nuclear receptor
translation regulator
phosphatase
transmembrane receptor
G-protein coupled receptor
ion channel
other
enzyme
transporter
kinase
transcription regulator
microRNA
cytokine
growth factor
peptidase
-5.847
3.347
B
Spleen
Rorc
Il1β
Il1f9
Cxcr2
*
***
Relative Expression
C
Liver
Relative Expression (x 10-4)
Relative Expression (x 10-2)
Relative Expression (x 10-3)
D
WT
Map3k1^ΔKD
Itgb7
Itgb21
Itgb1
