# Supplementary material for: T-Cell-Specific Deletion of Map3k1 Reveals the Critical Role for Mekk1 and Jnks in Cdkn1b-Dependent Proliferative Expansion
Source: Cell Rep. 2016 Jan 7;14(3):449–57. doi: 10.1016/j.celrep.2015.12.047 (PMC4733086; doi:10.1016/j.celrep.2015.12.047)
Supplement: Document S2. Article plus Supplemental Information [file mmc2.pdf]

Cell Reports

Report

T-Cell-Specific Deletion of *Map3k1* Reveals the Critical Role for Mekk1 and Jnks in *Cdkn1b*-Dependent Proliferative Expansion

Graphical Abstract

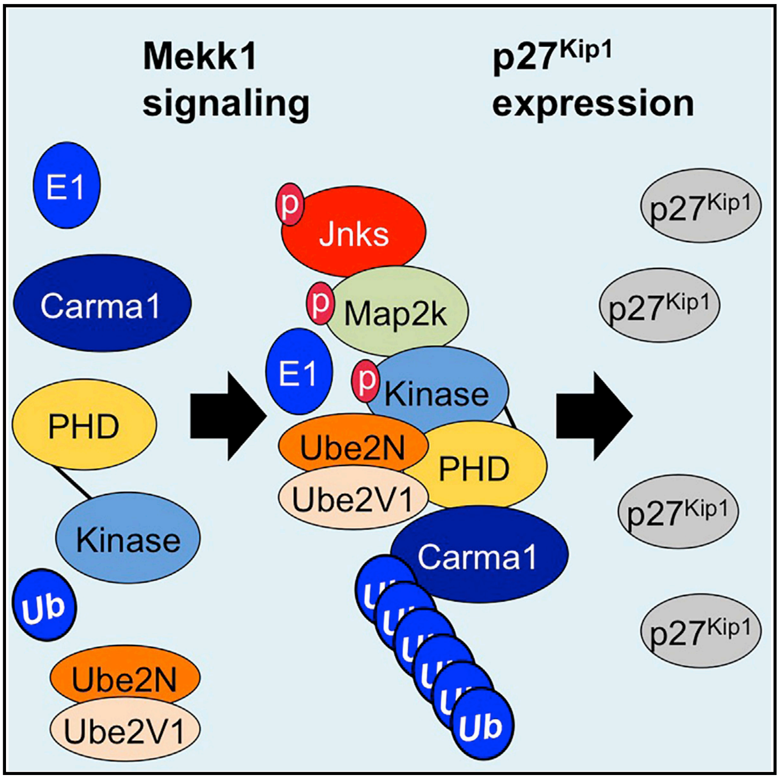

Authors

Tesha Suddason, Saba Anwar, Nikolaos Charlaftis, Ewen Gallagher

Correspondence

teshas7@yahoo.com (T.S.),
ewengallagher@outlook.com (E.G.)

In Brief

Suddason et al. use a T-cell-specific deletion of *Map3k1* to show that Mekk1 regulates TCR-dependent Jnk activation and *Cdkn1b* expression to drive proliferation in response to antigen.

Highlights

- iNKT cell expansion is aberrant in *Lck*$^{Cre/+}$ *Map3k1*$^{f/f}$ mice
- *Lck*$^{Cre/+}$ *Map3k1*$^{f/f}$ mice have enhanced liver damage in response to glycolipids
- Mekk1 regulates TCR-dependent Jnk activation
- Mekk1 regulates p27^{Kip1} expression to regulate proliferation

Accession Numbers

E-MTAB-1561

Suddason et al., 2016, Cell Reports *14*, 449–457
January 26, 2016 © 2016 The Authors.
http://dx.doi.org/10.1016/j.celrep.2015.12.047

CellPress

Cell Reports
Report

OPEN ACCESS
CellPress

T-Cell-Specific Deletion of *Map3k1* Reveals the Critical Role for Mekk1 and Jnks in *Cdkn1b*-Dependent Proliferative Expansion

Tesha Suddason,[1,*] Saba Anwar,[1] Nikolaos Charlaftis,[1] and Ewen Gallagher[1,*]
[1]Department of Medicine, Imperial College London, Du Cane Road, London W12 0NN, UK
*Correspondence: teshas7@yahoo.com (T.S.), ewengallagher@outlook.com (E.G.)
http://dx.doi.org/10.1016/j.celrep.2015.12.047
This is an open access article under the CC BY license (http://creativecommons.org/licenses/by/4.0/).

SUMMARY

MAPK signaling is important for T lymphocyte development, homeostasis, and effector responses. To better understand the role of Mekk1 (encoded by *Map3k1*) in T cells, we conditionally deleted *Map3k1* in *Lck*$^{Cre/+}$ *Map3k1*$^{f/f}$ mice, and these display larger iNKT cell populations within the liver, spleen, and bone marrow. Mekk1 signaling controls splenic and liver iNKT cell expansion in response to glycolipid antigen. *Lck*$^{Cre/+}$ *Map3k1*$^{f/f}$ mice have enhanced liver damage in response to glycolipid antigen. Mekk1 regulates Jnk activation in iNKT cells and binds and transfers Lys63-linked poly-ubiquitin onto Carma1. *Map3k1* is critical for the regulation of p27^{Kip1} (encoded by *Cdkn1b*).

INTRODUCTION

Mitogen-activated protein kinase (MAPK) kinase (MAP2K) kinases (MAP3Ks) are important regulators of IκB kinases (IKKs) and MAP2Ks (Dong et al., 2002; Ghosh and Karin, 2002; Kyriakis and Avruch, 2001; Raman et al., 2007; Suddason and Gallagher, 2015). Nineteen MAP3Ks are present in mammals, though their precise roles in regulating the immune system are not fully understood (Karin and Gallagher, 2005; Suddason and Gallagher, 2015). Mek kinase 1 (Mekk1) is unique in containing both a kinase domain and a plant homeodomain (PHD), that can bind transforming growth factor (Tgf)-β-activated kinase 1 (Tak1)-binding protein 1 (Tab1) and act as an E3 ubiquitin (Ub) ligase (Suddason and Gallagher, 2015; Charlaftis et al., 2014). *Map3k1*$^{\Delta KD}$ B cells regulate Jnk and p38 signaling from tumor necrosis factor (TNF) receptor family members (TNFRs) (Matsuzawa et al., 2008; Gallagher et al., 2007; Karin and Gallagher, 2009). Analysis of *Map3k1*$^{\Delta KD}$ T cells demonstrated that Mekk1 is an important regulator of T helper 2 (Th2) cytokine production by the Jnk-dependent activation of Itch (Enzler et al., 2009; Gallagher et al., 2006; Gao et al., 2004; Fang et al., 2002; Venuprasad et al., 2006). Moreover, an intact Mekk1 PHD motif is required for Itch phosphorylation following T cell receptor (TCR) signaling (Suddason and Gallagher, 2015; Charlaftis et al., 2014), though the means by which Mekk1 is recruited to the TCR remain to be clarified. *Map3k1*$^{\Delta KD}$ $CD8^+$ T cells display enhanced expansion in response to viruses, but the mechanism remains uncertain (Labuda et al., 2006). The analysis of the precise role of Mekk1 in T cells using *Map3k1*$^{\Delta KD}$ mice has been complicated by both B lymphocyte defects and also the partial lethality of *Map3k1*$^{\Delta KD}$ mice on the C57BL/6 background (Bonnesen et al., 2005; Gallagher et al., 2007).

T lymphocytes form a critical cellular component of the adaptive immune response and can be broadly subdivided into conventional and unconventional subtypes (Kronenberg and Gapin, 2002; von Boehmer, 1990). Of these, natural killer T (NKT) cells constitute a unique unconventional T cell population of the immune system (Kronenberg and Gapin, 2007). By contrast to conventional $CD4^+$ and $CD8^+$ T cells, which are reactive to major histocompatibility complex (MHC) class I- or II-associated peptides, NKT cells can recognize lipids in the context of CD1d molecules (Bendelac et al., 1995; Spada et al., 1998; Brossay et al., 1998). NKT cells may express a skewed range of TCR variable region genes and the natural killer (NK) cell marker NK1.1 (Sköld et al., 2000). NKT cells can be subdivided into three categories based on their reactivity to the glycolipid α-galactosylceramide (α-GalCer), TCR α chain diversity, and CD1d dependency. Type I invariant NKT (iNKT) cells have invariant Vα14-Jα18 TCR α chains and react to α-GalCer in a CD1d-dependent manner. Type II nonclassical NKT cells are unreactive to α-GalCer and have TCR α chain diversity but are CD1d dependent. NKT-like (or type III) cells are CD1d independent, unresponsive to α-GalCer, and possess diverse TCR α chains (Bendelac et al., 2007). Following TCR engagement by glycolipid presented by CD1d, iNKT cells undergo proliferative expansion and secrete cytokines (Kawano et al., 1997; Crowe et al., 2003; Parekh et al., 2005; Godfrey et al., 2010). Type I iNKT cells are abundant within the liver, where they are important regulators of inflammation and liver damage (Van Kaer et al., 2013).

Here, we investigate *Map3k1* by T-cell-specific and germline ablation in mice. *Map3k1* regulates iNKT cell proliferative expansion in response to glycolipid antigen. CARD-containing MAGUK protein 1 (Carma1), a TCR-associated scaffold protein, is a target for the Mekk1 PHD motif and provides a mechanism for Mekk1 recruitment to the TCR (Blonska and Lin, 2009; Rincón and Davis, 2007). Microarray gene profiling of *Map3k1*-deficient iNKT cells undergoing their clonal burst in response to glycolipid antigen identified *Cdkn1b* as a cell-cycle gene that is aberrantly expressed in *Map3k1*-deficient mice (Kiyokawa et al., 1996). The

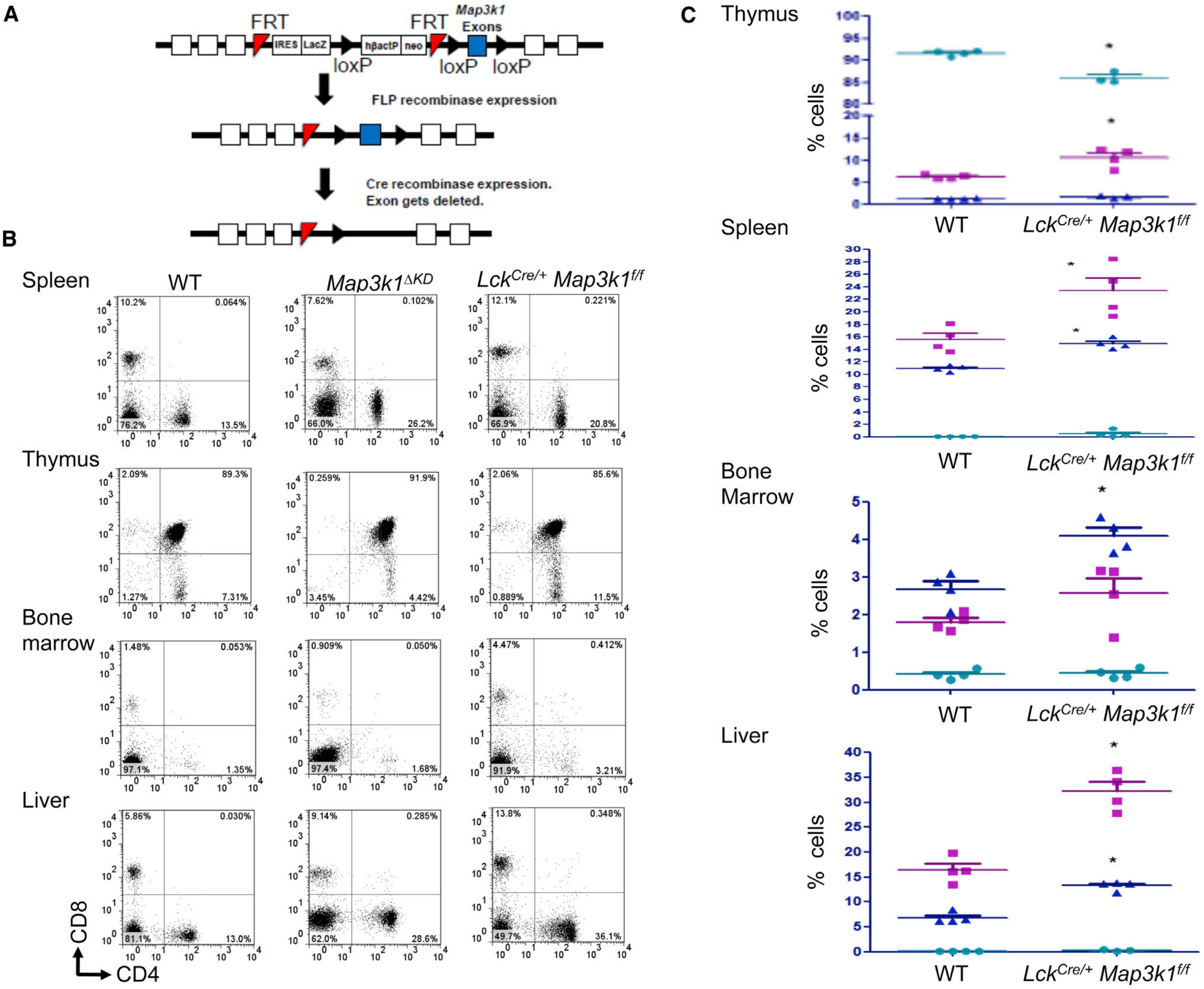

Figure 1. T Cell Development and Homeostasis within *Lck*$^{Cre/+}$ *Map3k1*$^{f/f}$ Mice

(A) A schematic diagram representing the construction of the *Map3k1*$^{f/f}$ allele.

(B) Thymocytes, splenocytes, bone marrow, and liver cells from WT, *Map3k1*$^{\Delta KD}$, and *Lck*$^{Cre/+}$ *Map3k1*$^{f/f}$ mice (all on the C57BL/6 background) were isolated, stained with anti-CD4 and anti-CD8 antibodies, and analyzed by flow cytometry as indicated. Data are representative of three independent experiments. Numbers in the profiles indicate the percentages of the gated populations.

(C) The average percentage (±SEM) of three cell populations $CD4^+CD8^+$, $CD4^+CD8^-$, and $CD4^-CD8^+$ cells from *Lck*$^{Cre/+}$ *Map3k1*$^{f/f}$ and WT mice from six independent experiments was statistically analyzed (green circle, $CD4^+CD8^+$; purple square, $CD4^+CD8^-$; blue triangle, $CD4^-CD8^+$), where appropriate, by two-tailed Student's t test (*$p \leq 0.05$; **$p \leq 0.01$; ***$p \leq 0.001$).

regulation of p27^{Kip1} by Mekk1 signaling provides a cell intrinsic molecular explanation for the altered proliferative expansion observed in both *Map3k1*$^{\Delta KD}$ and *Lck*$^{Cre/+}$ *Map3k1*$^{f/f}$ iNKT cells.

RESULTS

Map3k1 Regulates Conventional T Cells

Because *Map3k1*$^{\Delta KD}$ mice have both B cell defects and partial lethality on the C57BL/6 background, we generated *Lck*$^{Cre/+}$ *Map3k1*$^{f/f}$ mice (Figures 1A, S1A, and S1B) to better understand the roles of *Map3k1* in T cells (Gallagher et al., 2007; Bonnesen et al., 2005). Within the thymus of *Lck*$^{Cre/+}$ *Map3k1*$^{f/f}$ mice, there is a minor development defect with significantly fewer $CD4^+CD8^+$ double-positive thymocytes than WT but a significantly larger than WT population of $CD4^+$ single-positive thymocytes (Figures 1B and 1C; Chang et al., 2011; Charlaftis et al., 2014). However, the total number of thymocytes in *Map3k1*$^{\Delta KD}$ and *Lck*$^{Cre/+}$ *Map3k1*$^{f/f}$ mice is similar to WT (Table S1; Gao et al., 2004; Venuprasad et al., 2006; Labuda et al., 2006). Splenic $CD4^+$ T cells isolated from *Lck*$^{Cre/+}$ *Map3k1*$^{f/f}$ mice display an enhanced production of *Il4* following TCR crosslinking with anti-CD3 and anti-CD28 antibodies (Figure S1C), the

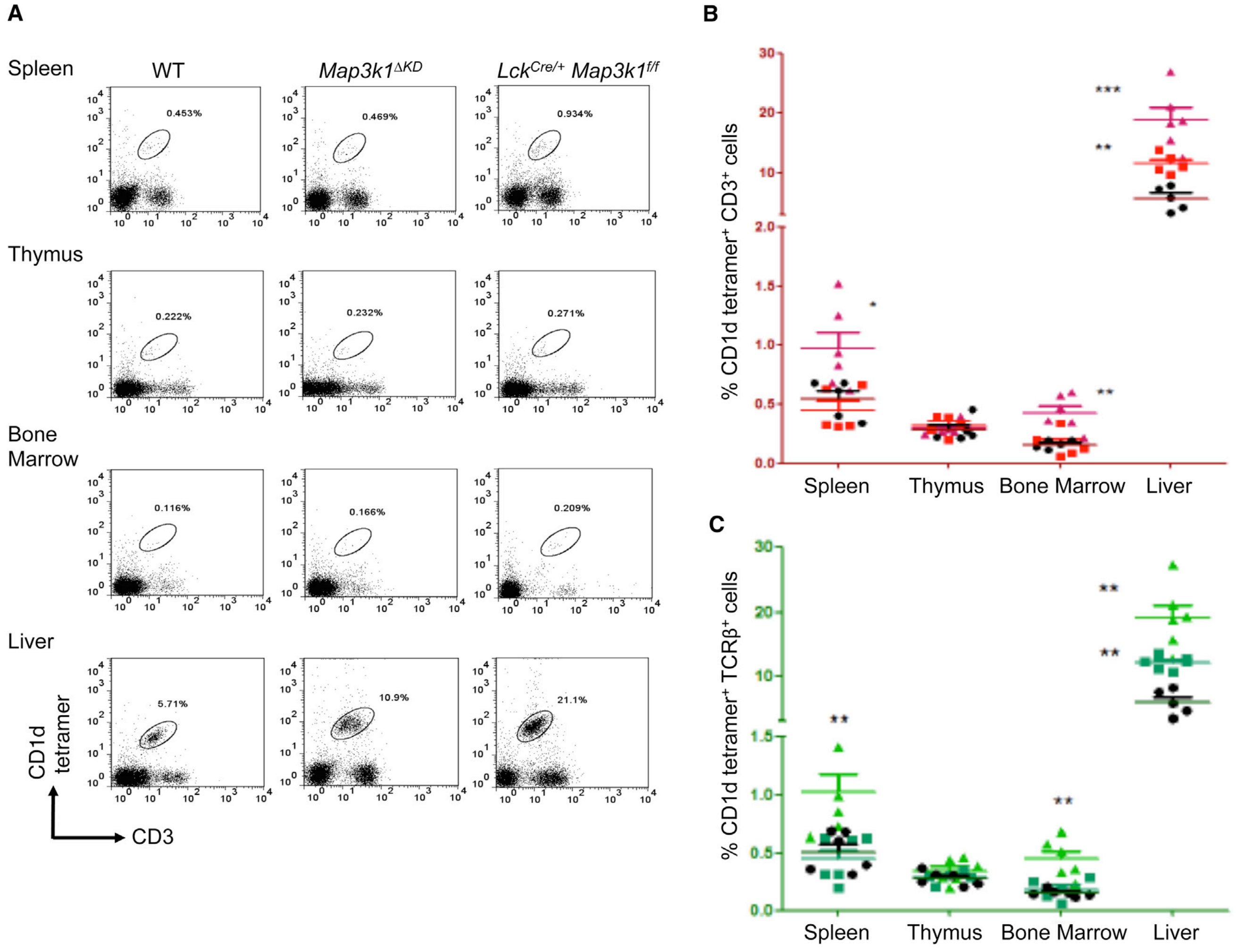

Figure 2. iNKT Cell Development and Homeostasis in *Map3k1$^{\Delta KD}$* and *Lck$^{Cre/+}$ Map3k1$^{f/f}$* Mice

(A) Cell suspensions were isolated from the spleen, thymus, bone marrow, and liver from WT, *Map3k1$^{\Delta KD}$*, and *Lck$^{Cre/+}$ Map3k1$^{f/f}$* mice, stained with CD1d tetramer and anti-CD3 antibody, and analyzed by flow cytometry as indicated. Data are representative of five independent experiments. Numbers in the profiles indicate the percentages of the gated populations.

(B) Statistical analysis of iNKT populations (CD1d tetramer$^+$CD3$^+$) within the spleen, thymus, bone marrow, and liver from WT, *Map3k1$^{\Delta KD}$*, and *Lck$^{Cre/+}$ Map3k1$^{f/f}$* mice. The average percentage (±SEM) of CD1d-tetramer and CD3-positive cells from five independent experiments is shown (black circle, WT; red square, *Map3k1$^{\Delta KD}$*; purple triangle, *Lck$^{Cre/+}$ Map3k1$^{f/f}$* mice). Statistical differences were analyzed by two-tailed Student's t test (*$p \leq 0.05$; **$p \leq 0.01$; ***$p \leq 0.001$).

(C) Statistical analysis of iNKT populations (CD1d tetramer$^+$TCRβ^+) within the spleen, thymus, bone marrow, and liver from WT, *Map3k1$^{\Delta KD}$*, and *Lck$^{Cre/+}$ Map3k1$^{f/f}$* mice. The average percentage (±SEM) of CD1d-tetramer and TCRβ-positive cells from five independent experiments is shown (black circle, WT; green square, *Map3k1$^{\Delta KD}$*; green triangle, *Lck$^{Cre/+}$ Map3k1$^{f/f}$* mice). Statistical differences were analyzed by two-tailed Student's t test (*$p \leq 0.05$; **$p \leq 0.01$; ***$p \leq 0.001$).

same Itch activation-dependent Th2 phenotype observed in *Map3k1$^{\Delta KD}$* CD4$^+$ T cells (Gao et al., 2004; Venuprasad et al., 2006). By contrast, $\gamma\delta$ T cells isolated from *Lck$^{Cre/+}$ Map3k1$^{f/f}$* or WT mice are not significantly different (Figure S1D; Maki et al., 1996). *Lck$^{Cre/+}$ Map3k1$^{f/f}$* mice display significantly more CD4$^+$ and CD8$^+$ T cells within the spleen and liver and a significantly larger CD8$^+$ T cell population within the bone marrow (Figures 1B and 1C; Chang et al., 2011; Charlaftis et al., 2014). No significant difference was detected in T cells isolated from the thymus, spleen, liver, or bone marrow between WT and *Lck$^{Cre/+}$* mice (Figure S1E; data not shown).

Map3k1 Regulates iNKT Cells

Lck$^{Cre/+}$ Map3k1$^{f/f}$ mice have significantly higher numbers of iNKT cells (CD1d tetramer$^+$CD3$^+$, CD1d tetramer$^+$TCRβ^+, or CD1d tetramer$^+$NK1.1$^+$) within the liver, spleen, and bone marrow relative to WT or *Lck$^{Cre/+}$* mice (Figures 2A–2C, S2A, and S2B; data not shown; Ansari et al., 2010). *Map3k1$^{\Delta KD}$* mice, which display a germline deletion of the *Map3k1* exons encoding the Mekk1 kinase domain (Gao et al., 2004), similarly displayed significantly higher numbers of iNKT cells (CD1d tetramer$^+$CD3$^+$, CD1d tetramer$^+$TCRβ^+, or CD1d tetramer$^+$NK1.1$^+$) in the liver (Figures 2B and 2C; data not shown). However,

OPEN
ACCESS
CellPress

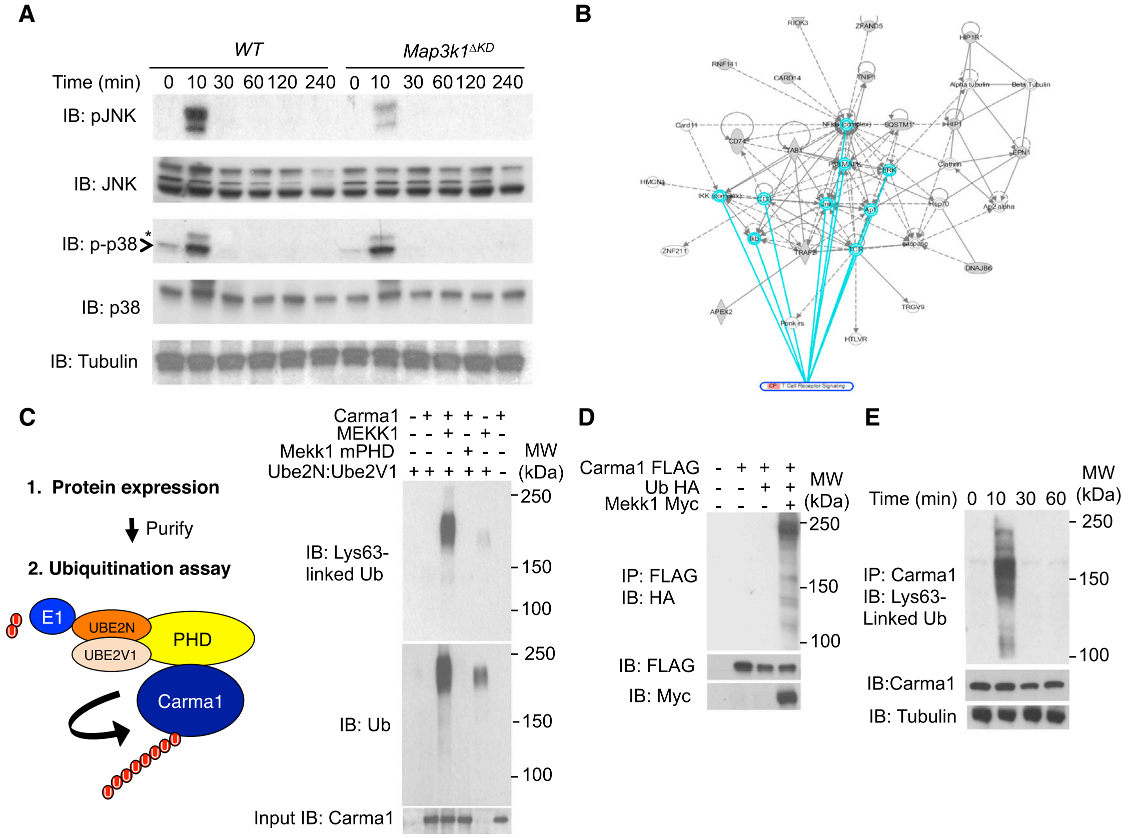

Figure 3. *Map3k1* Regulates Jnk Activation in iNKT Cells

(A) iNKT cells were isolated (four mice per experiment) and stimulated by TCR crosslinking with antibodies over a 240-min time course as indicated. Cell lysates were made and analyzed by IB with the indicated antibodies. Arrowhead indicates phospho-p38 and asterisk a non-specific band.

(B) IPA network diagram of TCR signaling to show the presence of the Mekk1 PHD substrate Carma within this pathway.

(C) In vitro ubiquitination assays using Mekk1 PHD, Mekk1 mPHD, Ube2N:Ube2V1, E1, Ub, and Carma1. Reactions were performed as indicated and analyzed by IB as indicated. A fraction of the ubiquitination reactions was taken pre-incubation, boiled, analyzed by IB as shown, and indicated as input.

(D) HEK293 cells were transfected with the indicated constructs. To detect in vivo ubiquitination, lysates were made under denaturing conditions for IP (Gallagher et al., 2007) and IB performed as indicated. Lysates were also made under non-denaturing conditions as a loading control and IB performed with the indicated antibodies.

(E) iNKT cells were isolated (four mice per experiment) and stimulated by TCR crosslinking with antibodies over a 60-min time course. To detect in vivo ubiquitination, lysates were made under denaturing conditions for IP (Gallagher et al., 2007) and IB performed as indicated. Lysates were also made under non-denaturing conditions as a loading control and IB performed with the indicated antibodies.

iNKT cell development in the thymus is normal for both *Map3k1*$^{\Delta KD}$ and *Lck*$^{Cre/+}$ *Map3k1*$^{f/f}$ mice (Figure S2C).

Mekk1 Regulates TCR-Dependent Jnk Activation in iNKT Cells

Map3k1$^{\Delta KD}$ iNKT cells were isolated and stimulated in vitro by TCR crosslinking with antibodies (Figure 3A; Gao et al., 2004; Nagaleekar et al., 2011). WT iNKT cells display a transient Jnk activation at 10 min that is significantly reduced in *Map3k1*$^{\Delta KD}$ iNKT cells following TCR crosslinking with antibodies (Figures 3A and S3A). Phosphorylation of c-Jun is similarly reduced in *Map3k1*$^{\Delta KD}$ iNKT and conventional cells following TCR cross-linking with antibodies (data not shown), but there is no significant defect in p38 activation (Figure S3B; Gao et al., 2004). Because Mekk1 binds and ubiquitinates proteins by its PHD motif, we analyzed a comprehensive Mekk1 PHD protein array screen by ingenuity pathway analysis (IPA) bioinformatics to identify hits that are important for TCR signaling and identified Carma1 as a possible Mekk1 PHD substrate (Figure 3B; Suddason and Gallagher, 2015; Charlaftis et al., 2014). The Mekk1 PHD binds and, in association with Ub-conjugating enzyme E2N (Ube2N), transfers Lys63-linked Ub chains onto Carma1 (Figures 3C and 3D). Mekk1 and Carma1 transiently co-purify from iNKT cells 10 min following TCR crosslinking (Figure S3C), and endogenous Carma1 is transiently ubiquitinated following TCR cross-linking with antibodies (Figures 3E and S3D).

Map3k1 Regulates Splenic and Liver iNKT Cell Expansion

To assess the role of Mekk1 signaling in iNKT cell responses to antigen, WT, *Map3k1*$^{\Delta KD}$, and *Lck*$^{Cre/+}$ *Map3k1*$^{f/f}$ mice were immunized with the iNKT cell TCR agonist α-GalCer (Figures 4

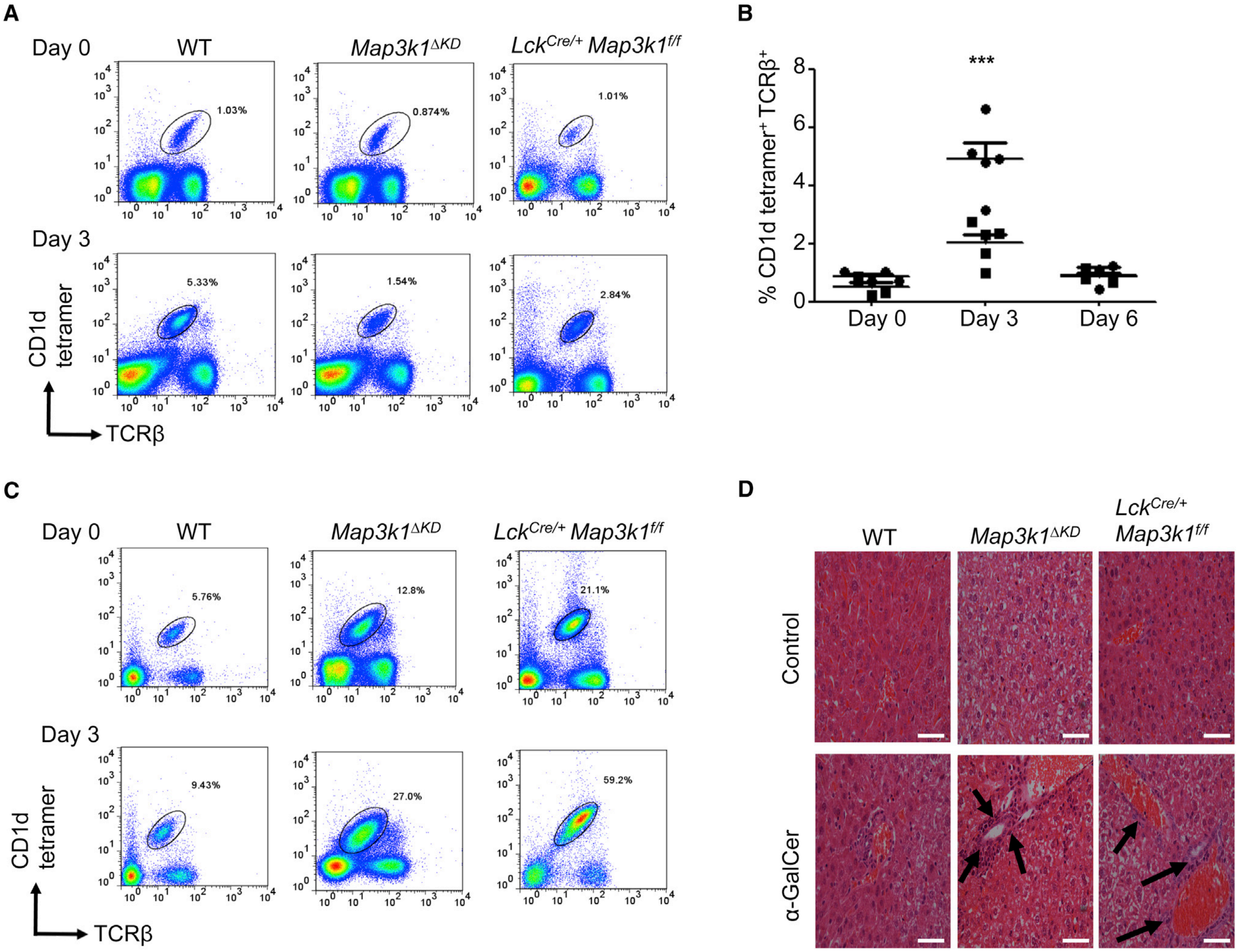

Figure 4. iNKT Cell Expansion in *Map3k1*-Deficient Mice

(A) WT, *Map3k1*$^{\Delta KD}$, and *Lck*$^{Cre/+}$ *Map3k1*$^{f/f}$ mice were i.p. injected with α-GalCer for 3 days. Splenocytes were harvested at days 0 and 3, stained with anti-TCRβ antibody and CD1d tetramer, and analyzed by flow cytometry as indicated. Data are representative of three independent experiments. Numbers in the profiles indicate the percentages of the gated populations.

(B) Statistical analysis of α-GalCer-dependent iNKT expansion at days 0, 3, and 6 in *Map3k1*$^{\Delta KD}$ mice. The average percentage (±SEM) of PBS-57-loaded CD1d tetramer$^+$ TCRβ$^+$ cells from five independent experiments is shown (black circle, WT; black square, *Map3k1*$^{\Delta KD}$ mice). Differences were analyzed by two-tailed Student's t test (*p ≤ 0.05; **p ≤ 0.01; ***p ≤ 0.001).

(C) Liver cells were harvested at days 0 and 3 from WT, *Map3k1*$^{\Delta KD}$, and *Lck*$^{Cre/+}$ *Map3k1*$^{f/f}$ mice following i.p. immunization with α-GalCer. Liver cells were stained with CD1d tetramer and anti-TCRβ antibodies and analyzed by flow cytometry as indicated. Data are representative of three independent experiments. Numbers in the profiles indicate the percentages of the gated populations.

(D) Representative H&E-stained liver sections were prepared from unstimulated (upper panels) and 3-day α-GalCer stimulated (lower panels) WT, *Map3k1*$^{\Delta KD}$, and *Lck*$^{Cre/+}$ *Map3k1*$^{f/f}$ mice (original magnification ×40; scale bar, 10 μM). Arrows indicate lymphocyte infiltration. Data are representative of three independent experiments (two mice per experiment).

and S4). Short-term stimulation of *Map3k1*$^{\Delta KD}$ and *Lck*$^{Cre/+}$ *Map3k1*$^{f/f}$ mice with α-GalCer lead to normal iNKT activation and cytokine production (Figure S4A; data not shown). By contrast, splenic iNKT cells from *Map3k1*$^{\Delta KD}$ and *Lck*$^{Cre/+}$ *Map3k1*$^{f/f}$ mice display significantly reduced long-term proliferative expansion following immunization with α-GalCer (Figures 4A, 4B, and S4B; data not shown). Conversely, liver iNKT cells from *Map3k1*$^{\Delta KD}$ and *Lck*$^{Cre/+}$ *Map3k1*$^{f/f}$ mice showed significantly enhanced long-term proliferative expansion following immunization with α-GalCer (Figures 4C, S4B, and S4C). Analysis of the livers from *Map3k1*$^{\Delta KD}$ and *Lck*$^{Cre/+}$ *Map3k1*$^{f/f}$ mice revealed significantly enhanced lymphocyte infiltration and liver damage following long-term immunization with α-GalCer relative to control mice (Figures 4D and S4D).

Mekk1 Controls iNKT Cell Proliferative Expansion by the Regulation of Jnk-Dependent p27^{Kip1} Expression

To understand the molecular basis underpinning the aberrant proliferative expansion of both *Lck*$^{Cre/+}$ *Map3k1*$^{f/f}$ and *Map3k1*$^{\Delta KD}$ iNKT cells, we analyzed global gene expression

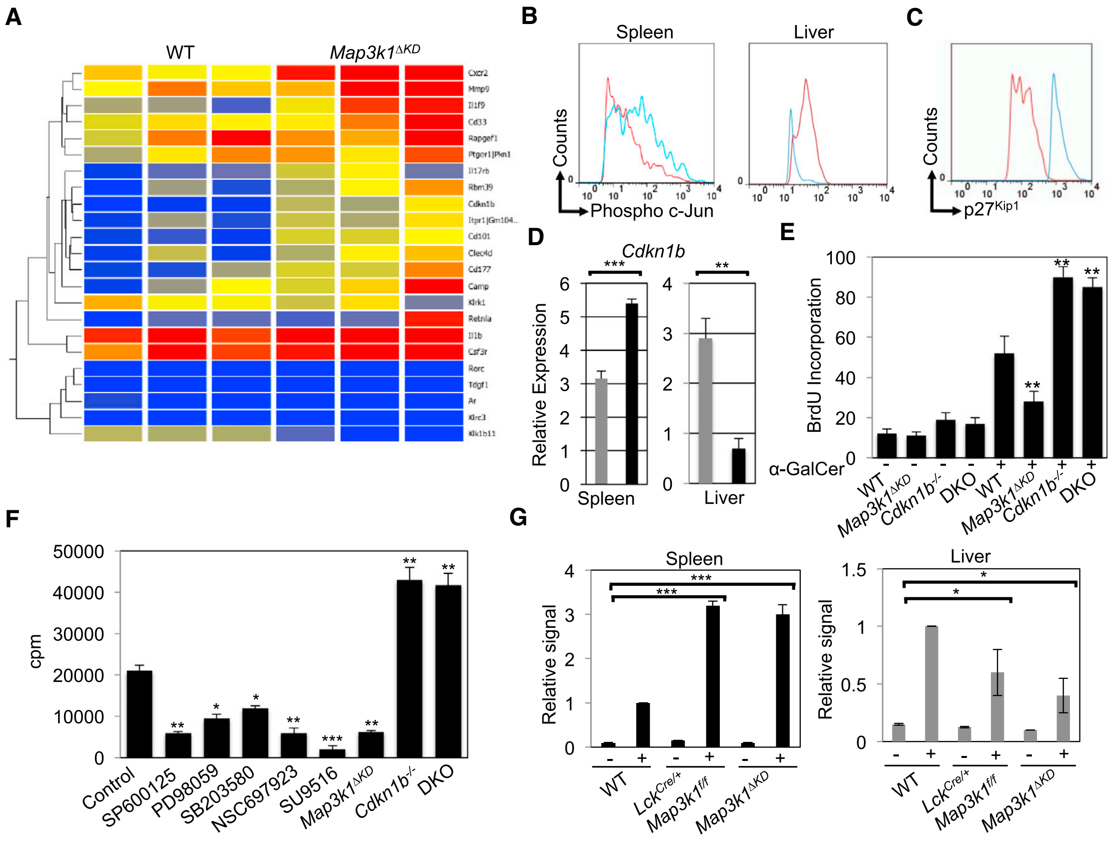

Figure 5. Mekk1 Signaling Controls p27^{Kip1} Expression to Regulate iNKT Cell Proliferation

(A) WT and *Map3k1$^{\Delta KD}$* mice were i.p. injected with α-GalCer for 3 days. RNA was isolated from WT and *Map3k1$^{\Delta KD}$* splenic iNKT cells, processed, and hybridized onto Affymetrix arrays. Bioinformatics analysis was performed, and a heatmap comparing gene hits between WT and *Map3k1$^{\Delta KD}$* iNKT cell microarray screens was constructed. The data are from three independent experiments (four mice per experiment).

(B) Splenic and liver iNKT cells were isolated from 3-day α-GalCer-immunized *Lck$^{Cre/+}$ Map3k1$^{f/f}$*, WT mice stained with anti-phospho c-Jun antibody, and flow cytometry performed as indicated (red line, WT; blue line, *Lck$^{Cre/+}$ Map3k1$^{f/f}$*). Data were representative of three independent experiments. Histograms show the phospho c-Jun present in the gated iNKT cell population.

(C) Splenic iNKT cells from 3-day α-GalCer-immunized *Lck$^{Cre/+}$ Map3k1$^{f/f}$* and WT mice were isolated and stained with anti-p27^{Kip1} antibody and flow cytometry performed as indicated (red line, WT; blue line, *Lck$^{Cre/+}$ Map3k1$^{f/f}$*). Data were representative of three independent experiments. Histogram shows the p27^{Kip1} present in the gated iNKT cell population.

(D) iNKT cells from the spleen and liver of WT and *Lck$^{Cre/+}$ Map3k1$^{f/f}$* mice were isolated 3 days post-i.p. injection with α-GalCer and their RNA analyzed by real-time PCR as indicated (gray square, WT; black square, *Lck$^{Cre/+}$ Map3k1$^{f/f}$*). The average relative expression (±SEM) of genes from three independent experiments was statistically analyzed, where appropriate, by two-tailed Student's t test (*p ≤ 0.05; **p ≤ 0.01; ***p ≤ 0.001).

(E) WT, *Map3k1$^{\Delta KD}$*, *Cdkn1b$^{-/-}$*, or *Map3k1$^{\Delta KD}$/Cdkn1b$^{-/-}$* (DKO) mice were treated with water containing BrdU and i.p. immunized with α-GalCer (day 3) or left unstimulated (day 0). Splenocytes were extracted and analyzed as indicated. Representative results (±SEM) from three quantitated iNKT proliferation experiments were statistically analyzed, where appropriate, by two-tailed Student's t test (*p ≤ 0.05; **p ≤ 0.01; ***p ≤ 0.001).

(F) WT, *Cdkn1b$^{-/-}$*, *Map3k1$^{\Delta KD}$*, or *Map3k1$^{\Delta KD}$/Cdkn1b$^{-/-}$* (DKO) iNKT cells were isolated and incubated in [^{3}H] thymidine containing media for 24 hr in the presence of DMSO (control), SP600125, PD98059, SB203580, NSC697923, or SU9516. The average cpm (±SEM) from three independent experiments was statistically analyzed, where appropriate, by two-tailed Student's t test (*p ≤ 0.05; **p ≤ 0.01; ***p ≤ 0.001).

(G) WT, *Map3k1$^{\Delta KD}$*, and *Lck$^{Cre/+}$ Map3k1$^{f/f}$* mice were immunized with α-GalCer for 3 days, splenic or liver iNKT cells isolated, and *Cdkn1b* ChIP performed with anti-phospho c-Jun antibody as indicated. The average relative signal (±SEM) from three independent experiments was statistically analyzed, where appropriate, by two-tailed Student's t test (*p ≤ 0.05; **p ≤ 0.01; ***p ≤ 0.001).

patterns following the long-term immunization of *Map3k1$^{\Delta KD}$* mice with α-GalCer (Figure 5A; Table S2). Bioinformatics analysis of the screened hits identified *Cdkn1b* (encoding p27^{Kip1}) as a *Map3k1*-dependent cell-cycle regulator (Figure S5A). *Lck$^{Cre/+}$ Map3k1$^{f/f}$* mice have enhanced long-term phospho-c-Jun in splenic iNKT cells but reduced phospho-c-Jun in liver iNKT cells, relative to WT following immunization with α-GalCer (Figure 5B). *Cdkn1b* and p27^{Kip1} expression is significantly enhanced in splenic *Map3k1$^{\Delta KD}$* and *Lck$^{Cre/+}$ Map3k1$^{f/f}$* iNKT cells, but *Cdkn1b* expression is significantly reduced in liver *Map3k1$^{\Delta KD}$* and *Lck$^{Cre/+}$ Map3k1$^{f/f}$* iNKT cells following long-term stimulation by α-GalCer (Figures 5C and 5D; data not shown). Similarly, other screened hits (including *Rorc*, *Il1β*, *Il1f9*, and *Cxcr2*) identified by global gene expression analysis

were verified by real-time PCR in splenic (Figure S5B; data not shown) and liver tissues (Figure S5C; data not shown). Integrin gene expression (*Itgb7*, *Itgb21*, and *Itgb1*) is equivalent between WT and *Map3k1*$^{\Delta KD}$ iNKT cells (Figure S5D). Whereas splenic iNKT cells from *Map3k1*$^{\Delta KD}$ and *Lck*$^{Cre/+}$ *Map3k1*$^{f/f}$ mice hypoproliferate, splenic *Cdkn1b*$^{-/-}$ iNKT cells hyperproliferate following long-term stimulation by α-GalCer (Figure 5E). iNKT cell proliferation in response to TCR crosslinking with antibodies is significantly reduced by chemical inhibition of Jnk, Ube2N, or cyclin-dependent kinases (CDKs) (Figure 5F). Splenic, in contrast to liver, *Map3k1*$^{\Delta KD}$ and *Lck*$^{Cre/+}$ *Map3k1*$^{f/f}$ iNKT cells display greater phosphorylation of c-Jun at the *Cdkn1b* promoter activator protein-1 (AP-1)-binding site following long-term stimulation by α-GalCer (Figure 5G; Khattar and Kumar, 2010).

DISCUSSION

We have shown, using *Lck*$^{Cre/+}$ *Map3k1*$^{f/f}$ mice, that Mekk1 has important roles in T cells. Thymic development is moderately skewed in *Lck*$^{Cre/+}$ *Map3k1*$^{f/f}$ mice, with reduced numbers of CD4$^+$CD8$^+$ double-positive thymocytes and enhanced numbers of CD4$^+$ single-positive thymocytes. Our findings differ from *Lck*$^{Cre/+}$ *Map3k7*$^{f/f}$ mice that display reduced numbers of CD4$^+$ and CD8$^+$ single-positive thymocytes (Wan et al., 2006) and *Lck*$^{Cre/+}$ *Map3k2*$^{-/-}$ *Map3k3* $^{f/f}$ mice that display normal thymic development (Chang et al., 2011). The CD4$^+$ and CD8$^+$ T cell populations are also larger within the spleen, bone marrow, and liver tissues of *Lck*$^{Cre/+}$ *Map3k1*$^{f/f}$ mice. *Map3k1*$^{\Delta KD}$ mice have elevated numbers of T cells in the liver, and analysis of the iNKT cell population revealed that these are significantly expanded in the liver of both *Map3k1*$^{\Delta KD}$ and *Lck*$^{Cre/+}$ *Map3k1*$^{f/f}$ mice. Two factors complicating the analysis of T cells using *Map3k1*$^{\Delta KD}$ mice have been the germline kinase domain mutation impacting B cells and partial embryonic lethality (Gao et al., 2004; Gallagher et al., 2007; Bonnesen et al., 2005). Bone marrow chimeras and in vitro assays using cells from *Map3k1*$^{\Delta KD}$ mice have previously demonstrated that B and T cell Mekk1 signaling defects are intrinsic (Gao et al., 2004; Venuprasad et al., 2006; Labuda et al., 2006; Gallagher et al., 2007). As such, conditional deletion of *Map3k1* in T cells using *Lck*$^{Cre/+}$ *Map3k1*$^{f/f}$ mice represents a significant refinement of the analysis of *Map3k1* in T cells.

TCR signaling leads to the rapid activation of MAPK and the phosphorylation of its downstream targets (e.g., c-Jun), and these can then initiate T cell effector responses (Su et al., 1994; Dong et al., 2002). More recently, E3 Ub ligase Itch was identified as a downstream Mekk1-signaling target that is phosphorylated by Jnk1 to induce a conformational change within the protein leading to Itch activation and canonical ubiquitination of Jun transcription factors (Gallagher et al., 2006; Gao et al., 2004; Fang et al., 2002; Venuprasad et al., 2006). As with conventional CD4$^+$ T cells, TCR signal transduction rapidly activates Jnk in iNKT cells and this is significantly reduced in *Map3k1*-deficent iNKT cells (Gao et al., 2004). Mekk1 transiently binds and ubiquitinates Carma1, a scaffold known to regulate Jnk activation, following TCR engagement, and this provides a mechanism of recruitment for Mekk1 to the TCR that differs from TNFRs or Tgf-β receptors (Suddason and Gallagher, 2015; Charlaftis et al., 2014; Gaide et al., 2002).

Our work identifies a role for *Map3k1* in the regulation of the iNKT cell proliferative expansion in response to glycolipid antigen. In order to identify the mechanism underpinning *Map3k1*-dependent iNKT cell expansion, we analyzed their global gene expression profile to identify *Cdkn1b* as a regulated target. The modulation of p27^{Kip1} expression in T cells by Mekk1 signaling represents a molecular mechanism that regulates T cell proliferation. We have identified increased iNKT cell infiltration into the liver and a higher degree of liver damage in *Map3k1*$^{\Delta KD}$ and *Lck*$^{Cre/+}$ *Map3k1*$^{f/f}$ mice. The aberrant iNKT cell expansion within the spleen and liver of *Map3k1*-deficient mice can be explained by altered c-Jun-dependent *Cdkn1b* expression. Our results reinforce the importance of Mekk1 signaling in T cells.

EXPERIMENTAL PROCEDURES

Gene Targeting of *Map3k1*

Map3k1 was targeted by insertion of a *FRT* site followed by a *LacZ* sequence and a *loxP* site into chromosome 13 upstream of the exons of the *Map3k1* gene (to generate the *Map3k1*^{f} allele; Skarnes et al., 2011). The first *loxP* site was followed by *neo* under the control of the human *B-actin* promoter, SV40 poly-A, a second *FRT* site, and a second *loxP* site. A third *loxP* site was inserted downstream of the *Map3k1* exons. *Map3k1*$^{+/f}$ ESCs (C57BL/6) were generated by standard procedures (Gossler et al., 1986) and genotyped by Southern blotting or genomic PCR (Ledermann, 2000; Gao et al., 2004; Charlaftis et al., 2014). Four independently generated *Map3k*$^{+/f}$ ESC clones were injected into blastocysts, and the resulting transgenics were genotyped by PCR (Gao et al., 2004; Charlaftis et al., 2014). Genomic PCR was carried out on mice biopsies using primers to detect the *Map3k1*-specific WT allele, the shorter mutant allele, and the recombinant allele (5′ to 3′ primers: TCGTGGTATCGTTATGCGCC; AATAGGCCACACGTTGACTGG; and CAACCCACGAAAGGAGGTTC; Charlaftis et al., 2014). *Map3k1*$^{+/f}$ mice were crossed with *ACTFLPe* mice (Jackson Laboratory) to initiate recombination at the *FRT* sites, deleting the *lacZ* and *neo* and resulting in offspring that contain a *Map3k1*$^{f/f}$ allele.

Cell Line and Cell Culture Conditions

HEK293 cells were maintained in DMEM (22320; Invitrogen) supplemented with 10% FBS (SH3007003; Thermo Scientific) and antibiotics in a humidified atmosphere at 37°C. Cells were passaged every 2 or 3 days when approaching full confluence (Charlaftis et al., 2014).

Transfection

HEK293 cells were plated in 6-well plates at a density of 1 × 10^6 cells per well. The following day, cells were transfected with Lipofectamine 2000 (11668-019; Invitrogen) or Jet Prime (114-07; Polyplus) transfection reagents according to the manufacturer's instructions. Cells were collected and lysed 48 hr later.

Tissue Preparation

Spleen and perfused liver tissues were mashed through a 70-μm and a 100-μm strainer, respectively, and resuspended in RPMI 1640 medium (Invitrogen) supplemented with 10% FBS (ThermoScientific). Following low-speed centrifugation, the splenic pellet was treated with red blood cell (RBC) lysis buffer (Sigma), washed, and resuspended in medium. The liver cell pellet was resuspended in 38% Percoll (GE Healthcare) and then centrifuged at 500 g for 20 min at room temperature. Cell pellets were treated with RBC lysis buffer, washed, and resuspended in medium.

Isolation of iNKT Cells

Mouse iNKT cells isolation from spleen or liver tissues was performed using PE-conjugated and α-GalCer-loaded CD1d tetramer (Nagaleekar et al., 2011). Tetrameric CD1d:α-GalCer cell complexes were purified using anti-PE

MicroBeads (Miltenyi Biotec). Residual B cells were depleted prior to iNKT cell enrichment using a CD45R (B220) MicroBead kit (Miltenyi Biotec), and the final iNKT cell purity obtained was greater than 95%.

Flow Cytometry

Cell staining with CD1d tetramer was followed by intracellular staining performed using a Fix/Perm kit (BD PharMingen). For intracellular staining, the cells were incubated with 50 μg/ml PMA (Sigma-Aldrich), 1 μM ionomycin (Sigma-Aldrich), and 10 μg/ml brefeldin A (Sigma-Aldrich) for 2 hr before processing. For 5-bromo-2-deoxyuridine (BrdU) labeling, mice were fed with BrdU (0.8 mg/ml) in drinking water supplemented with 5% (weight/volume) glucose 1 day prior to α-GalCer (2 μg) i.p. injection. Mice were treated with BrdU in drinking water for 3 days to study proliferation. Cells were surface stained, and BrdU staining was performed according to the manufacturer protocol (BD PharMingen BrdU Flow Kit). Cells were analyzed on a Cyan ADP (DakoCytomation) flow cytometer and further analyzed on a workstation using FlowJo software (TreeStar).

Immunoblotting, Immunoprecipitation, Real-Time PCR, and Chromatin Immunoprecipitation

iNKT cells were isolated by magnetic selection using MACS LS columns according to the manufacturers protocols (Miltenyi Biotech). Immunoblotting (IB) and immunoprecipitation (IP) were carried out as previously described (Gao et al., 2004). iNKT cell RNA was prepared with the RNeasy kit (QIAGEN), and total RNA (500 μg) was converted to cDNA using the High Capacity cDNA RT-Kit (Applied Biosystems). Real-time PCR was performed in triplicate with the appropriate gene primers (Invitrogen; Table S3) using SYBR Green (Applied Biosystems) and an ABI Prism 7700 Sequence Detector (Applied Biosystems). β-actin was used for normalization of results. Chromatin IP (ChIP) was performed using an EpiTect ChIP qPCR primer assay for mouse *Cdkn1b* kit (QIAGEN) according to the manufacturer instructions.

Ubiquitination Assays

Carma1 cDNA was overexpressed in HEK293 cells and IP performed with anti-FLAG antibody, washed extensively, and protein eluted. Subsequently, Carma1 was incubated for 1 hr at 37°C with the ubiquitination assay enzymes E1 (100 nM), Ube2N:Ub-conjugating enzyme E2 variant 1 (Ube2V1; 0.36 μM), Ub, and ATP, with or without WT Mekk1 PHD (100 ng) or Mekk1 mutant PHD (mPHD; 100 ng; Charlaftis et al., 2014). All ubiquitination assay reagents were from Boston Biochem.

Microarray and Bioinformatics Analysis

Total RNA from iNKT cells was reverse transcribed into biotinylated cRNA with an RNA amplification kit according to the manufacturer's instructions (Ambion). RNA quality was verified using a 2100 Bioanalyzer (Agilent Technologies). Samples were hybridized to Mouse Gene 1.0 ST arrays (Affymetrix; Charlaftis et al., 2014). Partek software was used according to the vendor protocols for data analysis, quality control, and for creating gene lists and scatterplots. GeneSpringX software was used according to the vendor protocols to generate heatmaps. Probes with a fold change of less than two were discarded. Probes were quantile normalized among all microarray data. Gene lists were uploaded into the IPA program (Ingenuity Systems) to generate relevant signaling networks and gene wheels according to the vendor instructions.

Liver Damage Assay

Mice were injected i.v. with 2 μg KRN7000. After 3 days, the livers were harvested, fixed in 4% paraformaldehyde, processed, and paraffin embedded. H&E staining was carried out on liver sections (4 μm). Slides were analyzed using an Olympus light microscope, and pictures were taken using Image Pro-Software at 40× magnification.

Statistical Analysis

Data were expressed as SEM. Statistical significance was determined by two-tailed Student's t test. All analyses were performed using GraphPad Prism 5 software (GraphPad).

ACCESSION NUMBERS

The accession number for the dataset reported in this paper is ArrayExpress: E-MTAB-1561.

SUPPLEMENTAL INFORMATION

Supplemental Information includes Supplemental Experimental Procedures, five figures, and three tables and can be found with this article online at http://dx.doi.org/10.1016/j.celrep.2015.12.047.

AUTHOR CONTRIBUTIONS

T.S., S.A., N.C., and E.G. performed experiments, analyzed data, and wrote the manuscript.

ACKNOWLEDGMENTS

Research was supported by grants from the Wellcome Trust (WT090939MA) and Cancer Research UK (C26616/A12679). We would like to thank Xin Lin (University of Texas, MD Anderson Cancer Center) for Carma1 constructs, Michael Karin (University of California at San Diego) for *Map3k1*$^{\Delta KD}$ mice, A. Wahid Ansari (Imperial College London) for collegiate FACS advice, and Shashi Prajapati (Biogen) for thoughts on the manuscript.

Received: December 5, 2014
Revised: September 22, 2015
Accepted: December 7, 2015
Published: January 7, 2016

REFERENCES

Ansari, A.W., Temblay, J.N., Alyahya, S.H., and Ashton-Rickardt, P.G. (2010). Serine protease inhibitor 6 protects iNKT cells from self-inflicted damage. J. Immunol. *185*, 877–883.

Bendelac, A., Lantz, O., Quimby, M.E., Yewdell, J.W., Bennink, J.R., and Brutkiewicz, R.R. (1995). CD1 recognition by mouse NK1+ T lymphocytes. Science *268*, 863–865.

Bendelac, A., Savage, P.B., and Teyton, L. (2007). The biology of NKT cells. Annu. Rev. Immunol. *25*, 297–336.

Blonska, M., and Lin, X. (2009). CARMA1-mediated NF-kappaB and JNK activation in lymphocytes. Immunol. Rev. *228*, 199–211.

Bonnesen, B., Orskov, C., Rasmussen, S., Holst, P.J., Christensen, J.P., Eriksen, K.W., Qvortrup, K., Odum, N., and Labuda, T. (2005). MEK kinase 1 activity is required for definitive erythropoiesis in the mouse fetal liver. Blood *106*, 3396–3404.

Brossay, L., Chioda, M., Burdin, N., Koezuka, Y., Casorati, G., Dellabona, P., and Kronenberg, M. (1998). CD1d-mediated recognition of an alpha-galactosylceramide by natural killer T cells is highly conserved through mammalian evolution. J. Exp. Med. *188*, 1521–1528.

Chang, X., Liu, F., Wang, X., Lin, A., Zhao, H., and Su, B. (2011). The kinases MEKK2 and MEKK3 regulate transforming growth factor-β-mediated helper T cell differentiation. Immunity *34*, 201–212.

Charlaftis, N., Suddason, T., Wu, X., Anwar, S., Karin, M., and Gallagher, E. (2014). The MEKK1 PHD ubiquitinates TAB1 to activate MAPKs in response to cytokines. EMBO J. *33*, 2581–2596.

Crowe, N.Y., Uldrich, A.P., Kyparissoudis, K., Hammond, K.J., Hayakawa, Y., Sidobre, S., Keating, R., Kronenberg, M., Smyth, M.J., and Godfrey, D.I. (2003). Glycolipid antigen drives rapid expansion and sustained cytokine production by NK T cells. J. Immunol. *171*, 4020–4027.

Dong, C., Davis, R.J., and Flavell, R.A. (2002). MAP kinases in the immune response. Annu. Rev. Immunol. *20*, 55–72.

Enzler, T., Chang, X., Facchinetti, V., Melino, G., Karin, M., Su, B., and Gallagher, E. (2009). MEKK1 binds HECT E3 ligase Itch by its amino-terminal

RING motif to regulate Th2 cytokine gene expression. J. Immunol. *183*, 3831–3838.

Fang, D., Elly, C., Gao, B., Fang, N., Altman, Y., Joazeiro, C., Hunter, T., Copeland, N., Jenkins, N., and Liu, Y.C. (2002). Dysregulation of T lymphocyte function in itchy mice: a role for Itch in TH2 differentiation. Nat. Immunol. *3*, 281–287.

Gaide, O., Favier, B., Legler, D.F., Bonnet, D., Brissoni, B., Valitutti, S., Bron, C., Tschopp, J., and Thome, M. (2002). CARMA1 is a critical lipid raft-associated regulator of TCR-induced NF-kappa B activation. Nat. Immunol. *3*, 836–843.

Gallagher, E., Gao, M., Liu, Y.C., and Karin, M. (2006). Activation of the E3 ubiquitin ligase Itch through a phosphorylation-induced conformational change. Proc. Natl. Acad. Sci. USA *103*, 1717–1722.

Gallagher, E., Enzler, T., Matsuzawa, A., Anzelon-Mills, A., Otero, D., Holzer, R., Janssen, E., Gao, M., and Karin, M. (2007). Kinase MEKK1 is required for CD40-dependent activation of the kinases Jnk and p38, germinal center formation, B cell proliferation and antibody production. Nat. Immunol. *8*, 57–63.

Gao, M., Labuda, T., Xia, Y., Gallagher, E., Fang, D., Liu, Y.C., and Karin, M. (2004). Jun turnover is controlled through JNK-dependent phosphorylation of the E3 ligase Itch. Science *306*, 271–275.

Ghosh, S., and Karin, M. (2002). Missing pieces in the NF-kappaB puzzle. Cell *109* (*Suppl*), S81–S96.

Godfrey, D.I., Stankovic, S., and Baxter, A.G. (2010). Raising the NKT cell family. Nat. Immunol. *11*, 197–206.

Gossler, A., Doetschman, T., Korn, R., Serfling, E., and Kemler, R. (1986). Transgenesis by means of blastocyst-derived embryonic stem cell lines. Proc. Natl. Acad. Sci. USA *83*, 9065–9069.

Karin, M., and Gallagher, E. (2005). From JNK to pay dirt: jun kinases, their biochemistry, physiology and clinical importance. IUBMB Life *57*, 283–295.

Karin, M., and Gallagher, E. (2009). TNFR signaling: ubiquitin-conjugated TRAFfic signals control stop-and-go for MAPK signaling complexes. Immunol. Rev. *228*, 225–240.

Kawano, T., Cui, J., Koezuka, Y., Toura, I., Kaneko, Y., Motoki, K., Ueno, H., Nakagawa, R., Sato, H., Kondo, E., et al. (1997). CD1d-restricted and TCR-mediated activation of valpha14 NKT cells by glycosylceramides. Science *278*, 1626–1629.

Khattar, E., and Kumar, V. (2010). Mitogenic regulation of p27(Kip1) gene is mediated by AP-1 transcription factors. J. Biol. Chem. *285*, 4554–4561.

Kiyokawa, H., Kineman, R.D., Manova-Todorova, K.O., Soares, V.C., Hoffman, E.S., Ono, M., Khanam, D., Hayday, A.C., Frohman, L.A., and Koff, A. (1996). Enhanced growth of mice lacking the cyclin-dependent kinase inhibitor function of p27(Kip1). Cell *85*, 721–732.

Kronenberg, M., and Gapin, L. (2002). The unconventional lifestyle of NKT cells. Nat. Rev. Immunol. *2*, 557–568.

Kronenberg, M., and Gapin, L. (2007). Natural killer T cells: know thyself. Proc. Natl. Acad. Sci. USA *104*, 5713–5714.

Kyriakis, J.M., and Avruch, J. (2001). Mammalian mitogen-activated protein kinase signal transduction pathways activated by stress and inflammation. Physiol. Rev. *81*, 807–869.

Labuda, T., Christensen, J.P., Rasmussen, S., Bonnesen, B., Karin, M., Thomsen, A.R., and Odum, N. (2006). MEK kinase 1 is a negative regulator of virus-specific CD8(+) T cells. Eur. J. Immunol. *36*, 2076–2084.

Ledermann, B. (2000). Embryonic stem cells and gene targeting. Exp. Physiol. *85*, 603–613.

Maki, K., Sunaga, S., Komagata, Y., Kodaira, Y., Mabuchi, A., Karasuyama, H., Yokomuro, K., Miyazaki, J.I., and Ikuta, K. (1996). Interleukin 7 receptor-deficient mice lack gammadelta T cells. Proc. Natl. Acad. Sci. USA *93*, 7172–7177.

Matsuzawa, A., Tseng, P.H., Vallabhapurapu, S., Luo, J.L., Zhang, W., Wang, H., Vignali, D.A., Gallagher, E., and Karin, M. (2008). Essential cytoplasmic translocation of a cytokine receptor-assembled signaling complex. Science *321*, 663–668.

Nagaleekar, V.K., Sabio, G., Aktan, I., Chant, A., Howe, I.W., Thornton, T.M., Benoit, P.J., Davis, R.J., Rincon, M., and Boyson, J.E. (2011). Translational control of NKT cell cytokine production by p38 MAPK. J. Immunol. *186*, 4140–4146.

Parekh, V.V., Wilson, M.T., Olivares-Villagómez, D., Singh, A.K., Wu, L., Wang, C.R., Joyce, S., and Van Kaer, L. (2005). Glycolipid antigen induces long-term natural killer T cell anergy in mice. J. Clin. Invest. *115*, 2572–2583.

Raman, M., Chen, W., and Cobb, M.H. (2007). Differential regulation and properties of MAPKs. Oncogene *26*, 3100–3112.

Rincón, M., and Davis, R.J. (2007). Choreography of MAGUKs during T cell activation. Nat. Immunol. *8*, 126–127.

Skarnes, W.C., Rosen, B., West, A.P., Koutsourakis, M., Bushell, W., Iyer, V., Mujica, A.O., Thomas, M., Harrow, J., Cox, T., et al. (2011). A conditional knockout resource for the genome-wide study of mouse gene function. Nature *474*, 337–342.

Sköld, M., Faizunnessa, N.N., Wang, C.R., and Cardell, S. (2000). CD1d-specific NK1.1+ T cells with a transgenic variant TCR. J. Immunol. *165*, 168–174.

Spada, F.M., Koezuka, Y., and Porcelli, S.A. (1998). CD1d-restricted recognition of synthetic glycolipid antigens by human natural killer T cells. J. Exp. Med. *188*, 1529–1534.

Su, B., Jacinto, E., Hibi, M., Kallunki, T., Karin, M., and Ben-Neriah, Y. (1994). JNK is involved in signal integration during costimulation of T lymphocytes. Cell *77*, 727–736.

Suddason, T., and Gallagher, E. (2015). A RING to rule them all? Insights into the Map3k1 PHD motif provide a new mechanistic understanding into the diverse roles of Map3k1. Cell Death Differ. *22*, 540–548.

Van Kaer, L., Parekh, V.V., and Wu, L. (2013). Invariant natural killer T cells as sensors and managers of inflammation. Trends Immunol. *34*, 50–58.

Venuprasad, K., Elly, C., Gao, M., Salek-Ardakani, S., Harada, Y., Luo, J.L., Yang, C., Croft, M., Inoue, K., Karin, M., and Liu, Y.C. (2006). Convergence of Itch-induced ubiquitination with MEKK1-JNK signaling in Th2 tolerance and airway inflammation. J. Clin. Invest. *116*, 1117–1126.

von Boehmer, H. (1990). Developmental biology of T cells in T cell-receptor transgenic mice. Annu. Rev. Immunol. *8*, 531–556.

Wan, Y.Y., Chi, H., Xie, M., Schneider, M.D., and Flavell, R.A. (2006). The kinase TAK1 integrates antigen and cytokine receptor signaling for T cell development, survival and function. Nat. Immunol. *7*, 851–858.

Cell Reports

Supplemental Information

T-Cell-Specific Deletion of *Map3k1* Reveals the Critical Role for Mekk1 and Jnks in *Cdkn1b*-Dependent Proliferative Expansion

Tesha Suddason, Saba Anwar, Nikolaos Charlaftis, and Ewen Gallagher

Supplementary information

Supplementary materials

Mice

Map3k1$^{\Delta KD}$ mice were generated as previously described (Gao et al., 2004), and *Cdkn1b*$^{-/-}$ mice (The Jackson Laboratory) were maintained in accordance with the vendor's protocols. To produce mice with a T cell-specific *Map3k1* deletion, *Map3k1*$^{f/f}$ homozygote mice were crossed with *Lck*^{Cre} mice (Jackson Laboratory) (Hennet et al., 1995). All strains of mice were present on the C57BL/6 background, and bred and maintained under pathogen free conditions in accordance with the guidelines of Imperial College London and the Home Office (London, UK).

Reagents and antibodies

PBS-57-loaded CD1d tetramers were provided by the National Institutes of Health (NIH) Tetramer Facility (Atlanta, USA). KRN7000 (Enzo Life Sciences), an α-GalCer analogue, was used to stimulate iNKT cells. Anti-APC TCRβ (H57-597) (eBioscience), anti-FITC CD3ε (145-2C11) (eBioscience), anti-FITC NK1.1 (PK136) (eBioscience), anti-APC CD44 (IM7) (eBioscience), anti-FITC CD8 (53-6.7) (eBioscience), anti-PE Cy7-CD4 (GK1.5) (eBioscience), anti-FITC γδ TCR (eBioscience) anti-PE Cy7-IFN-γ (BVD6-24G2) (Cell Signaling), anti-phospho p38 (15) (Cell Signaling), anti-phospho JNK (26) (Cell Signaling), anti-p27^{Kip1} antibody (JZA0211031) (R&D Systems), anti-HA (32-6700) (Invitrogen), anti-Myc (R950-25) (Invitrogen), anti-p38 (9212) (Cell Signaling), anti-phospho p38 (9211) (Cell Signaling), anti-JNK (9252) (Cell Signaling), anti-c-Jun (60A8) (Cell Signaling), anti-phospho c-Jun (Ser73) (D47G9) (Cell Signaling), anti-phospho c-Jun (Ser73) (D47G9) XP® Rabbit mAb (Alexa Fluor® 488 Conjugate) (Cell Signaling), anti-

CARD11 (sc-20458) (Cell Signaling), anti-mouse Ub (13-1600) (Invitrogen), anti-Lys63-linked Ub (5621) (Cell Signaling), anti-phospho ERK (M8159) (Sigma), anti-ERK (M5670) (Sigma) and anti-Tubulin (T5168) (Sigma) antibodies were used according to the manufacturers protocols. SP600125 (IC_{50} 40 nM JNK inhibitor, Sigma), PD98059 (IC_{50} 2 μM MEK inhibitor, Sigma), SB203580 (IC_{50} 0.5 μM p38 inhibitor, Sigma), SU9516 (IC_{50} 200 nM CDK inhibitor, Santa Cruz) and NSC697923 (IC_{50} 745 nM Ube2N inhibitor, Millipore) were used according to the manufacturers protocols. Mekk1 constructs were generated and utilized as previously described (Charlaftis et al., 2014).

Supplementary figure and table legends

Figure S1. T cell-specific deletion of *Map3k1*, related to figure 1. (A) Schematic illustrating the genomic PCR genotyping strategy for the floxed *Map3k1* allele. (B) Genomic PCR of the floxed *Map3k1* allele (WT band of 266 bp, mutant band of 113 bp and recombinant band of 408 bp) using DNA prepared from WT, *Map3k1*$^{f/+}$ and *Map3k1*$^{f/f}$ mice. (C) $CD4^{+}$ T cells were isolated from WT, *Map3k1*$^{\Delta KD}$ and *Lck*$^{Cre/+}$ *Map3k1*$^{f/f}$ mice and incubated with anti-CD3 and anti-CD28 antibodies. Cytokine mRNA level was quantitated after 24 hrs by real-time PCR and the relative amount of *Il4* mRNA to WT cells was given an arbitrary value of 1. The average relative amount (± SEM) from 3 independent experiments was statistically analyzed by two-tailed Student's t test (*, $p \leq 0.05$; **, $p \leq 0.01$; ***, $p \leq 0.001$). (D) Intraepithelial lymphocytes were isolated from the small intestine of WT and *Lck*$^{Cre/+}$ *Map3k1*$^{f/f}$ mice, stained with anti-γδ TCR and anti-CD3 antibodies and analyzed by flow cytometry as indicated. Data is representative of 3 independent experiments. Numbers in the profiles indicate the percentages of the gated populations. (E) Thymocytes,

splenocytes and liver cells from WT and *Lck*$^{Cre/+}$ mice were isolated, stained with anti-CD4 and anti-CD8 antibodies and analyzed by flow cytometry as indicated. Data is representative of 3 independent experiments. Numbers in the profiles indicate the percentages of the gated populations.

Figure S2. *Map3k1* regulates iNKT cells, related to figure 2. (A) Cell suspensions were isolated from the spleen, thymus, bone marrow and liver from WT and *Lck*$^{Cre/+}$ mice, stained with CD1d tetramer and anti-CD3 antibodies and analyzed by flow cytometry as indicated. Data is representative of 3 independent experiments. Numbers in the profiles indicate the percentages of the gated populations. (B) Splenocyte, thymocyte, bone marrow and liver cells from WT, *Map3k1*$^{\Delta KD}$ and *Lck*$^{Cre/+}$*Map3k1*$^{f/f}$ mice were isolated, stained with PBS-57-loaded CD1d tetramer and anti-NK1.1 antibody and analyzed by flow cytometry as indicated. Data is representative of 3 independent experiments. Numbers in the profiles indicate the percentages of the gated populations. (C) Statistical analysis of the iNKT population (CD1d tetramer^{+}TCRβ^{+}) within the thymus from WT, *Map3k1*$^{\Delta KD}$ and *Lck*$^{Cre/+}$ *Map3k1*$^{f/f}$ mice. The average percentage (± SEM) of CD1d-tetramer and TCRβ positive cells from 5 independent experiments is shown (• WT, ■ *Map3k1*$^{\Delta KD}$ and ▲ *Lck*$^{Cre/+}$ *Map3k1*$^{f/f}$ mice). Statistical differences were analyzed by two-tailed Student's t test (*, $p \leq 0.05$; **, $p \leq 0.01$; ***, $p \leq 0.001$).

Figure S3. Analysis of Mekk1 signaling in iNKT cells, related to figure 3. (A) Quantitation of phospho-Jnk activation in Fig. 3A. Mean intensity for each experimental point was measured by densitometry. (B) Quantitation of phospho-p38 activation in Fig. 3A. Mean intensity for each experimental point was measured by

densitometry. (C) iNKT cells were isolated (4 mice per experiment) and stimulated by TCR crosslinking with antibodies over a 60 mins timecourse. Lysates were made under non-denaturing conditions and IB and IP performed as indicated. (D) A model illustrating Mekk1-dependent TCR signaling in iNKT cells.

Figure S4. Role of *Map3k1* in the iNKT cell response to glycolipid antigen, related to figure 4. (A) Short-term iNKT cell expansion in WT and $Lck^{Cre/+}$ $Map3k1^{f/f}$ mice. WT (-) and $Lck^{Cre/+}$ $Map3k1^{f/f}$ (-) mice were i.p. immunized with α-GalCer for 2 hrs, splenocytes isolated and stained with CD1d tetramer, anti-IFN-γ (top panel), anti-IL-4 (middle panel) and anti-CD69 (bottom panel) antibodies and analyzed by flow cytometry as indicated. Data is representative of three independent experiments (2 mice per experiment). Histograms show the IFN-γ (top panel), IL-4 (middle panel) and CD69 (bottom panel) present in the gated iNKT cell population. (B) WT, $Map3k1^{\Delta KD}$ and $Lck^{Cre/+}$ $Map3k1^{f/f}$ mice were treated with water containing BrdU and i.p. immunized with α-GalCer for 3 days or left unimmunized. iNKT cells were isolated as above (■ spleen and ■ liver) and analyzed by flow cytometry for BrdU incorporation. Representative results from 3 quantitated iNKT proliferation experiments were statistically analyzed, where appropriate, by two-tailed Student's t test (*, p ≤ 0.05; **, p ≤ 0.01; ***, p ≤ 0.001). (C) Statistical analysis of α-GalCer-dependent liver iNKT expansion at days 0 and 3 in *Map3k1*-deficient mice. The average percentage (± SEM) of PBS-57-loaded CD1d tetramer^{+} TCRβ^{+} cells from 5 independent experiments is shown (■ WT, ■ $Map3k1^{\Delta KD}$ and ■ $Lck^{Cre/+}$ $Map31^{f/f}$ mice). Differences were analyzed by two-tailed Student's t test (*, p ≤ 0.05; **, p ≤ 0.01; ***, p ≤ 0.001). (D) Representative H&E stained liver sections were prepared from unstimulated (upper panels) and 3-day α-GalCer stimulated (lower panels) WT

and $Lck^{Cre/+}$ mice (original magnification x40, scale bar = 10 µM). Data is representative of 3 independent experiments (2 mice per experiment).

Figure S5. Mekk1 signaling regulates *Cdkn1b* expression and iNKT cell proliferative expansion, related to figure 5. (A) The bioinformatics gene wheel analyzes global gene expression hits from $Map3k1^{\Delta KD}$ iNKT cells and indicates their cellular and molecular function. Color intensity indicates the range of expression from an upregulation of 3.347 to a downregulation of -5.847. (B) WT and $Lck^{Cre/+}$ $Map3k1^{f/f}$ mice were 3-day immunized with α-GalCer (■ WT and ■ $Lck^{Cre/+}$ $Map3k1^{f/f}$), RNA was extracted from their splenic iNKT cells, reverse transcribed and real-time PCR performed. The average relative expression (± SEM) of genes from 3 independent experiments was statistically analyzed, where appropriate, by two-tailed Student's t test (*, $p \leq 0.05$; **, $p \leq 0.01$; ***, $p \leq 0.001$). (C) WT and $Lck^{Cre/+}$ $Map3k1^{f/f}$ mice were 3-day immunized with α-GalCer, RNA was extracted from liver iNKT cells, RNA was reverse transcribed and real-time PCR performed (■ WT and ■ $Lck^{Cre/+}$ $Map3k1^{f/f}$). The average relative expression (± SEM) of genes from 3 independent experiments was statistically analyzed, where appropriate, by two-tailed Student's t test (*, $p \leq 0.05$; **, $p \leq 0.01$; ***, $p \leq 0.001$). (D). RNA was isolated from WT and $Map3k1^{\Delta KD}$ iNKT cells, processed and hybridized onto Affymetrix arrays. Bioinformatics analysis was performed on integrin gene expression as indicated and a heat map comparing gene hits between WT and $Map3k1^{\Delta KD}$ iNKT cell microarray screens was constructed. The data is from 3 independent experiments (4 mice per experiment).

Table S1. Analysis of thymocytes in *Map3k1* mutant mice, related to figure 1. Total numbers of thymocytes from WT and $Lck^{Cre/+}$ $Map3k1^{f/f}$ mice were counted and percentages of subpopulations were quantitated by FACS analysis (Gao et al., 2004).

	**WT**	**$Lck^{Cre/+}$ $Map3k1^{f/f}$ mice**
**Total cell number ($\times 10^6$)**	172 ±49	175 ±51
**$CD4^+CD8^+$(%)**	91.65±0.30	86.1±0.808
**$CD4^-CD8^+$(%)**	6.28±0.204	10.56±1.073
**$CD4^+CD8^-$(%)**	10.93±0.052	14.4±0.221

Table S2. iNKT cell affymetrix microarray hits from *Map3k1*-deficient versus control mice undergoing a clonal burst (2-fold or more changes are listed), related to figure 5.

ID	Fold Change	Entrez Gene Name	Location	Type(s)
Klrc3	2.799	killer cell lectin-like receptor subfamily C, member 2	Plasma Membrane	other
Ar	2.397	androgen receptor	Nucleus	ligand-dependent nuclear receptor
Klrk1	2.266	killer cell lectin-like receptor subfamily K, member 1	Plasma Membrane	transmembrane receptor
Klk1b11	2.186	kallikrein-related peptidase 3	Extracellular Space	peptidase
Cd101	-2.008	CD101 molecule	Plasma Membrane	other
Cd177	-2.039	CD177 molecule	Cytoplasm	other
Clec4d	-2.042	C-type lectin domain family 4, member D	Plasma Membrane	other
Il1r2	-2.054	interleukin 1 receptor, type II	Plasma Membrane	transmembrane receptor
Rorc	-2.066	RAR-related orphan receptor C	Nucleus	ligand-dependent nuclear receptor
Cd33	-2.078	CD33 antigen	Plasma Membrane	other
Tdgf1	-2.110	teratocarcinoma-derived growth factor 1	Extracellular Space	growth factor
Il1b	-2.152	interleukin 1, beta	Extracellular Space	cytokine
Camp	-2.230	cathelicidin antimicrobial peptide	Cytoplasm	other
Csf3r	-2.246	colony stimulating factor 3 receptor (granulocyte)	Plasma Membrane	transmembrane receptor
Cxcr2	-2.257	chemokine (C-X-C motif) receptor 2	Plasma Membrane	G-protein coupled receptor
Il17rb	-2.345	interleukin 17 receptor B	Plasma Membrane	transmembrane receptor
Mmp9	-2.345	matrix metallopeptidase 9 (gelatinase B, 92kDa gelatinase, 92kDa type IV collagenase)	Extracellular Space	peptidase
Cdkn1b	-2.386	cyclin-dependent kinase inhibitor 1B (p27, Kip1)	Nucleus	kinase
Retnla	-2.628	resistin like alpha	Extracellular Space	other
Rbm39	-2.672	RNA binding motif protein 39	Nucleus	transcription regulator
Il1f9	-2.699	interleukin 36, gamma	Extracellular Space	cytokine
Rapgef1	-2.914	Rap guanine nucleotide exchange factor (GEF) 1	Cytoplasm	other
Ptger1	-3.025	prostaglandin E receptor 1 (subtype EP1), 42kDa	Plasma Membrane	G-protein coupled receptor

Table S3. Listing of the primers used for real-time PCR, related to experimental procedures.

	5' to 3'	
	Forward Primer	Reverse Primer
β-actin	GTCGACAACGGCTCCGGCATGT	TCCCACCATCACACCCTGGTGCCTA
Ar	TGAGCCAGGAGTGGTGTGTGC	AAGTTGCGGAAGCCAGGCAAGG
Camp	AGGAACAGGGGGTGGTGAAGCA	AGAAGTCCAGCCAGCCGGGAA
Cdkn1b	GCCAGACGTAAACAGCTCCGAATTA	AGAGGCAGATGGTTTAAGAGTGCCT
CD101	AGTTTCTGCTAAGTTCAGCATCGGC	TTCCTTGGTCGGGGCGCTTG
CD177	CCTTGCTACCCTGTGTCCCAGC	GGCAACCCTCGCTAACCTCGC
CD33	GGGGAGGCAACGGTCAAGCTC	CCTGCTGATGAGCCTGTGTATGGAA
Clec4d	ACCCGACATCCCCAACTGATCCC	GCAGGTCCAAGTACCTCCTGTAGC
Csf3r	ACCCCATGGATGTTGCCCCC	CTTCCTGCAGGGGCGTTGGC
Cxcr2	ATCTTCGCTGTCGTCCTTGT	AGCCAAGAATCTCCGTAGCA
Il17rb	GGAGGCAAGGAAGGAGCACGA	CGGCCCCATCTCGGCGATTT
Il1b	CCCTGCAGCTGGAGAGTGTGGA	TGTGCTCTGCTTGTGAGGTGCTG
Il1f9	CCACAGAGTAACCCCAGTCAGCG	TTCCACCTGTCCGGGTGTGGT
Il1r2	CGGGTCAAAGGAACAACCACGGA	CGGTCACACGGCCTCTTGGG
klk1b11	CACCCACGAAATTCCAAACCCCAG	CCGGCGTATTGGGTTTGCCACA
klrc3	AGACAGTGAAGAGGAGCAGGACTTT	TCTGCTGTGAGACCAGAAGCTGAC
klrk1	TCTGCTCAGAGATGAGCAAATGCC	GCCAAGGCTATAGCAAGGACTCGAA
Mmp9	TGCCCTACCCGAGTGGACGC	AGCCCAGTGCATGGCCGAAC
Ppbp	TCCTTGTTGCGCTGGCTCCC	GTGTGGCTATCACTTCCACATCAGC
Ptger1	ACATGCATGGGGTGGAGCAGC	TATCAGTGGCCAAGAGGGCCAG
Retnla	CCTGCCCTGCTGGGATGACT	GGGCAGTGGTCCAGTCAACGA
Rorc	TTCCCACTTCCTCAGCGCCC	TGGGTGGCAGCTTGGCTAGGA
Tdgf1	TCGCAAAGAGCACTGTGGGTC	AGTGGTCGTCACAGACGGCG

Supplementary references

CHARLAFTIS, N., SUDDASON, T., WU, X., ANWAR, S., KARIN, M. & GALLAGHER, E. 2014. The MEKK1 PHD ubiquitinates TAB1 to activate MAPKs in response to cytokines. *EMBO J,* 33**,** 2581-96.

GAO, M., LABUDA, T., XIA, Y., GALLAGHER, E., FANG, D., LIU, Y. C. & KARIN, M. 2004. Jun turnover is controlled through JNK-dependent phosphorylation of the E3 ligase Itch. *Science,* 306**,** 271-5.

HENNET, T., HAGEN, F. K., TABAK, L. A. & MARTH, J. D. 1995. T-cell-specific deletion of a polypeptide N-acetylgalactosaminyl-transferase gene by site-directed recombination. *Proc Natl Acad Sci U S A,* 92**,** 12070-4.

Fig S1

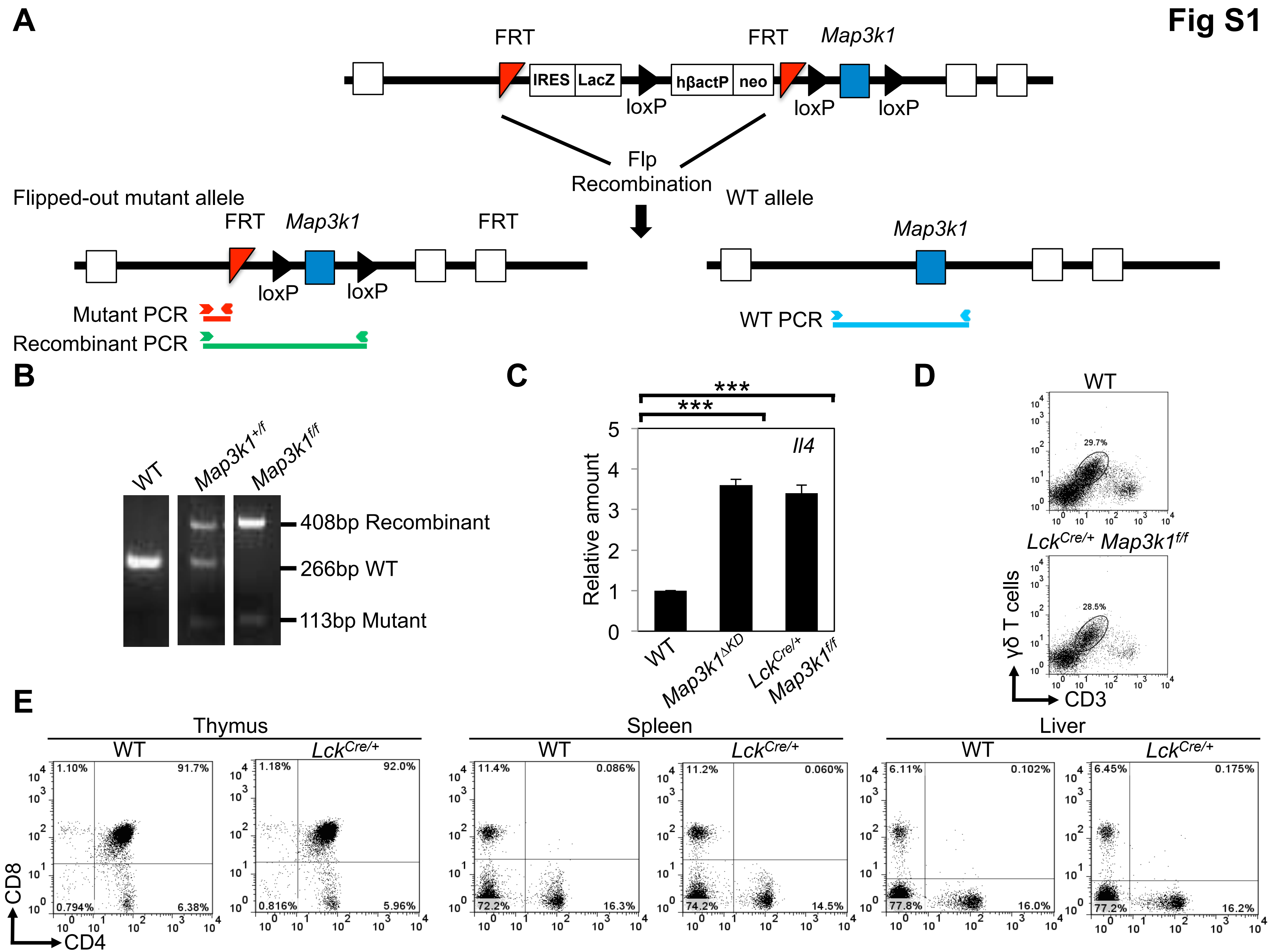

A
FRT
FRT
Map3k1
IRES
LacZ
hβactP
neo
loxP
loxP
loxP
Flp
Recombination
Flipped-out mutant allele
FRT
Map3k1
FRT
loxP
loxP
Mutant PCR
Recombinant PCR
WT allele
Map3k1
WT PCR
B
WT
Map3k1+/f
Map3k1f/f
408bp Recombinant
266bp WT
113bp Mutant
C
***
***
Il4
Relative amount
WT
Map3k1ΔKD
LckCre/+ Map3k1f/f
D
WT
29.7%
LckCre/+ Map3k1f/f
28.5%
γδ T cells
CD3
E
Thymus
Spleen
Liver
WT
LckCre/+
1.10%
91.7%
0.794%
6.38%
1.18%
92.0%
0.816%
5.96%
11.4%
0.086%
72.2%
16.3%
11.2%
0.060%
74.2%
14.5%
6.11%
0.102%
77.8%
16.0%
6.45%
0.175%
77.2%
16.2%
CD8
CD4

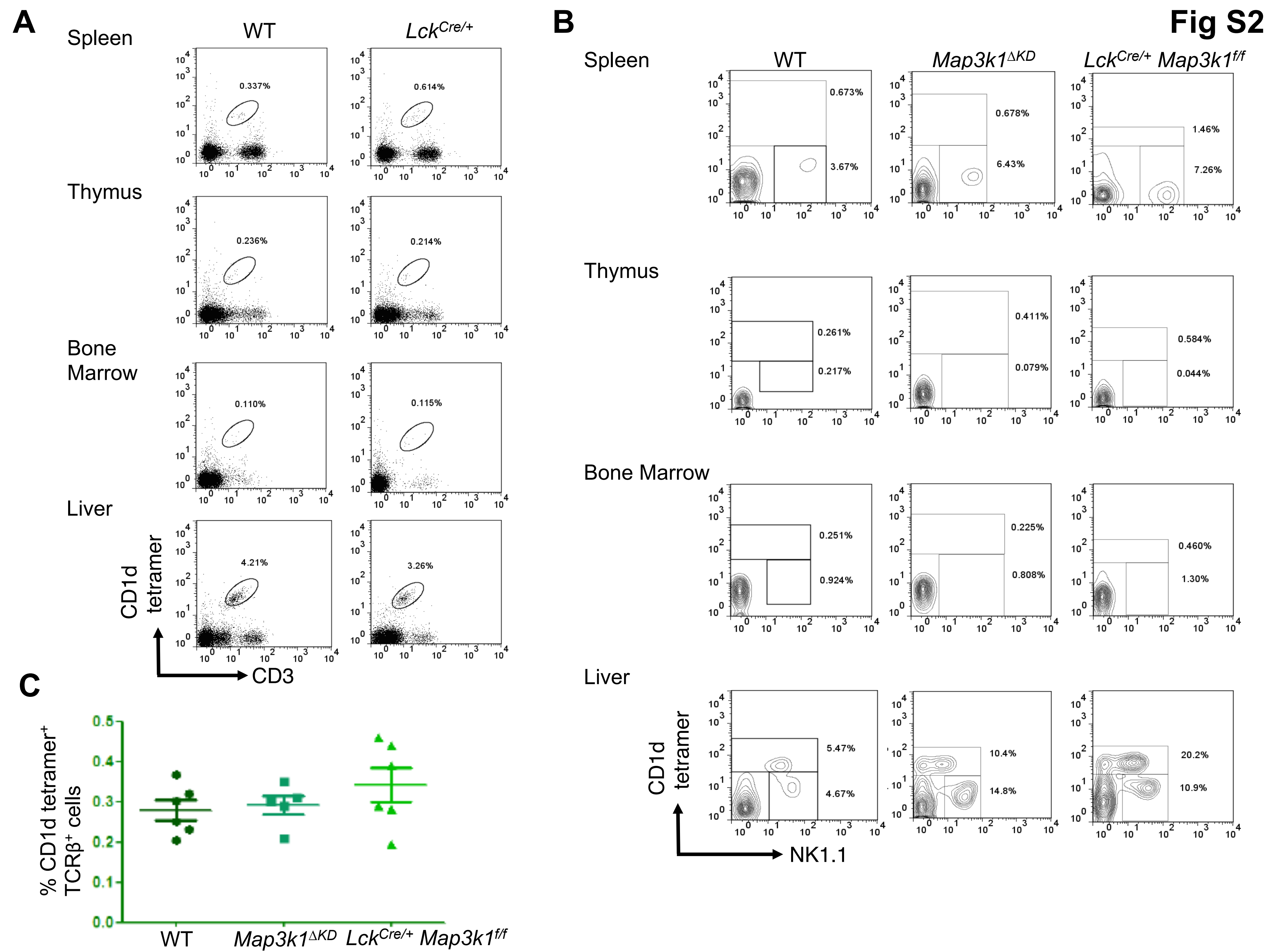
Fig S2
A
Spleen
Thymus
Bone Marrow
Liver
WT
LckCre/+
0.337%
0.614%
0.236%
0.214%
0.110%
0.115%
4.21%
3.26%
CD1d tetramer
CD3
B
WT
Map3k1ΔKD
LckCre/+ Map3k1f/f
0.673%
3.67%
0.678%
6.43%
1.46%
7.26%
0.261%
0.217%
0.411%
0.079%
0.584%
0.044%
0.251%
0.924%
0.225%
0.808%
0.460%
1.30%
5.47%
4.67%
10.4%
14.8%
20.2%
10.9%
NK1.1
C
% CD1d tetramer+ TCRβ+ cells
0.0
0.1
0.2
0.3
0.4
0.5

Fig S3

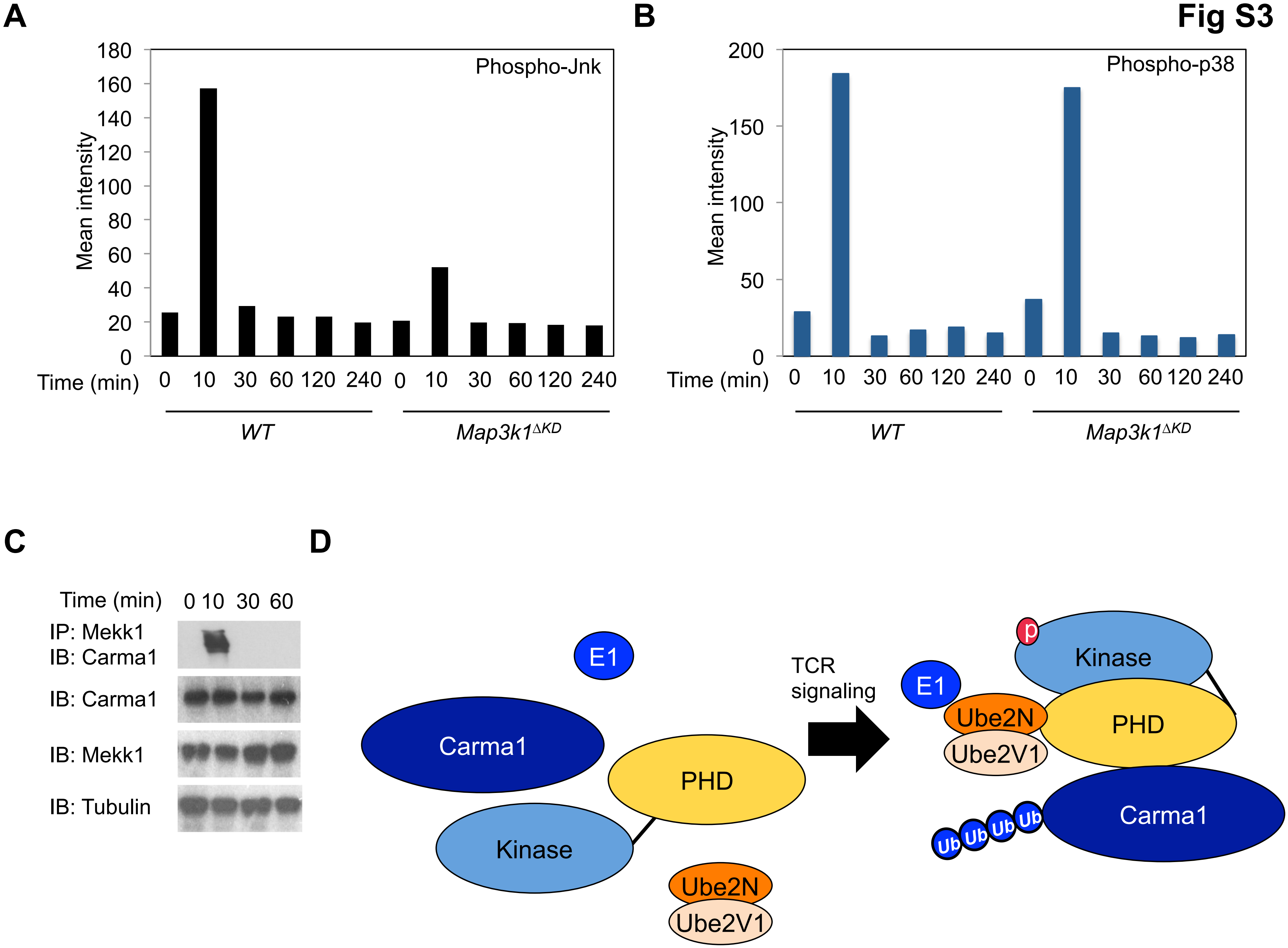
A
180
160
140
120
100
80
60
40
20
0
Mean intensity
Phospho-Jnk
Time (min) 0 10 30 60 120 240 0 10 30 60 120 240
WT
Map3k1ΔKD
B
200
150
100
50
0
Mean intensity
Phospho-p38
Time (min) 0 10 30 60 120 240 0 10 30 60 120 240
WT
Map3k1ΔKD
C
Time (min) 0 10 30 60
IP: Mekk1
IB: Carma1
IB: Carma1
IB: Mekk1
IB: Tubulin
D
E1
Carma1
PHD
Kinase
Ube2N
Ube2V1
TCR
signaling
p
Kinase
E1
Ube2N
PHD
Ube2V1
Carma1
Ub Ub Ub Ub

Fig S4

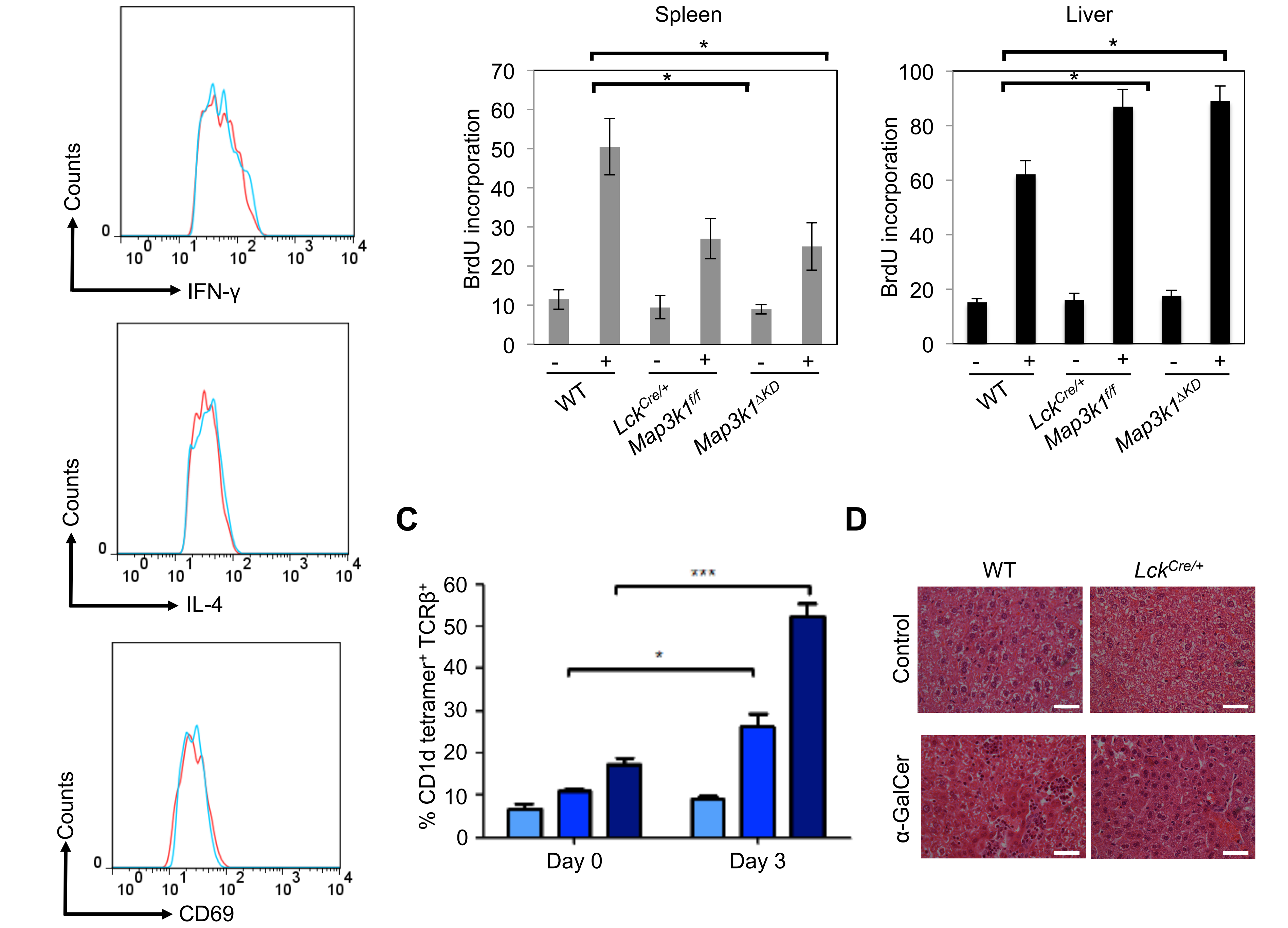
A
Counts
0
IFN-γ
Counts
0
IL-4
Counts
0
CD69
B
Spleen
Liver
BrdU incorporation
70
60
50
40
30
20
10
0
100
80
60
40
20
0
*
*
- + - + - +
WT
LckCre/+ Map3k1f/f
Map3k1ΔKD
C
% CD1d tetramer+ TCRβ+
***
*
Day 0
Day 3
D
WT
LckCre/+
Control
α-GalCer

Fig S5

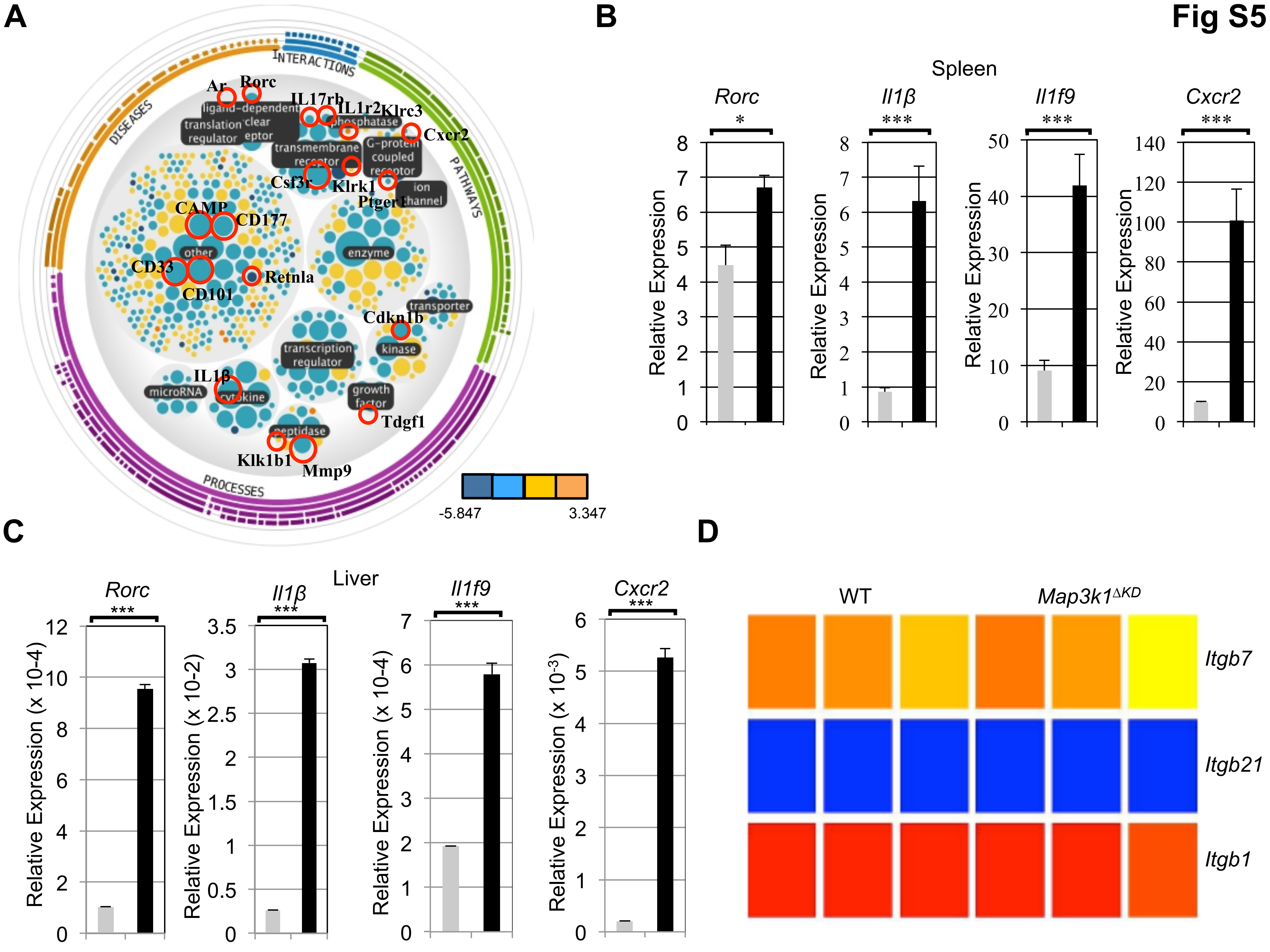
A
B
C
D
INTERACTIONS
DISEASES
PATHWAYS
PROCESSES
Ar
Rorc
IL17rb
IL1r2
Klrc3
Cxcr2
Csf3r
Klrk1
Ptger1
CAMP
CD177
CD33
CD101
Retnla
Cdkn1b
IL1β
Tdgf1
Klk1b1
Mmp9
ligand-dependent nuclear receptor
translation regulator
phosphatase
transmembrane receptor
G-protein coupled receptor
ion channel
other
enzyme
transporter
kinase
transcription regulator
microRNA
cytokine
growth factor
peptidase
-5.847
3.347
Spleen
Rorc
Il1β
Il1f9
Cxcr2
*
***
Relative Expression
Liver
Relative Expression (x 10-4)
Relative Expression (x 10-2)
Relative Expression (x 10-3)
WT
Map3k1ΔKD
Itgb7
Itgb21
Itgb1
